# *Agrostis* and *Podagrostis* (Agrostidinae, Poaceae) from páramos of Boyacá, Colombia: synoptic taxonomy including a key to Colombian species

**DOI:** 10.3897/phytokeys.151.50538

**Published:** 2020-06-24

**Authors:** Steven P. Sylvester, Lia E. Cuta-Alarcon, William J. Bravo-Pedraza, Robert J. Soreng

**Affiliations:** 1 College of Biology and the Environment, Nanjing Forestry University, Long Pan Road No. 159, Nanjing, 210037, China Nanjing Forestry University Nanjing China; 2 Royal Botanic Gardens, Kew, Richmond, Surrey, TW9 3AE, UK Royal Botanic Gardens Kew United Kingdom; 3 Grupo Sistemática Biológica, Herbario UPTC, Escuela de Biología, Facultad de Ciencias, Universidad Pedagógica y Tecnológica de Colombia, Avenida Central del Norte 39-115, Tunja-Boyacá, Colombia Universidad Pedagógica y Tecnológica de Colombia Tunja Colombia; 4 Department of Botany, National Museum of Natural History, Smithsonian Institution, Washington DC 20560, USA Smithsonian Institution Washington United States of America

**Keywords:** Andes, Gramineae, grasses, grassland, identification key, Neotropics, South America, taxonomy

## Abstract

We present taxonomic notes, including updated species descriptions and images, for the nine species of *Agrostis* and one species of *Podagrostis* found in páramos of Departamento Boyacá, Colombia (*A.boyacensis*, *A.breviculmis*, *A.capillaris*, *A.foliata*, A.cf.imberbis, *A.mertensii*, *A.perennans* s.l., *A.stolonifera*, *A.tolucensis*, *Podagrostistrichodes*). Agrostiscf.imberbis, previously known from austral South America, is newly recorded for Colombia, *A.capillaris* is a new regional record for Boyacá, and the name *Agrostisstuebelii* is lectotypified. We include keys in English and Spanish to distinguish the 15 species of *Agrostis* and two species of *Podagrostis* that are cited as occurring in Colombia.

## Abstract (es)

Se presentan notas taxonómicas, además de descripciones e imágenes actualizadas, para las nueve especies de *Agrostis* y una especie de *Podagrostis* encontradas en los páramos del departamento de Boyacá, Colombia (*A.boyacensis*, *A.breviculmis*, *A.capillaris*, *A.foliata*, A.cf.imberbis, *A.mertensii*, *A.perennans* s.l., *A.stolonifera*, *A.tolucensis*, *Podagrostistrichodes*). Agrostiscf.imberbis previamente conocido de Sudamerica austral, se presenta como un nuevo registro para Colombia y *A.capillaris* se presenta como un nuevo registro regional para Boyacá con el nombre de *Agrostisstuebelii* también lectotipificado. Se incluyen claves en inglés y español para distinguir las 15 especies de *Agrostis* y dos especies de *Podagrostis* citadas para Colombia.

## Introduction

The grass genera *Agrostis* L. and *Podagrostis* (Griseb.) Scribn. & Merr. belong to tribe Poeae R.Br., subtribe Agrostidinae Fr., with molecular data supporting their close relationship (Saarela et al. 2017). They both share numerous morphological characteristics, including paniculate, single-flowered laterally-compressed spikelets that disarticulate above the glumes, while lacking characteristics such as notably pubescent calluses and prominent rachilla prolongations that define the related genera once placed in *Calamagrostis* Adans. s.l. (Peterson et al. 2019; Sylvester et al. 2019a). Indeed, *Podagrostis* was originally described as a section of *Agrostis* and considered to be limited to just one species in Austral South America and three species from North America (Soreng 2003). Recent taxonomic research in Colombia has, however, discovered two new species of *Podagrostis* for South America, with *P.colombiana* Sylvester & Soreng being found from the páramos of the Sierra Nevada de Santa Marta, Colombia (Sylvester et al. 2019b), and the common High-Andean grass *Agrostistrichodes* (Kunth) Roem. & Schult. being transferred to *Podagrostis* (Sylvester et al. 2020). Aside from these studies, the only other published taxonomic research related to the genus *Agrostis* in Colombia is mostly limited to type protologues and checklists, some with a single voucher cited (Luteyn 1999; Soreng et al. 2003, 2003 and onwards; Giraldo-Cañas 2011, 2013; Giraldo-Cañas et al. 2016), with no taxonomic revisions or keys to the species existing for the genus in Colombia. Palacio et al. (in press) recently described the new species *Agrostislaegaardiana* A.M. Molina & Rúgolo, from Ecuadorian and Colombian páramos, and provided a brief key to distinguish morphologically similar species with condensed spikelike panicles from these habitats. With this, coupled with the new record of Agrostiscf.imberbis Phil. described herein and not including the new combination *Podagrostistrichodes* (Kunth) Sylvester & Soreng (Sylvester et al. 2020), Colombia is believed to hold 15 species of *Agrostis*, including three possible endemics, and three exotic species (Giraldo-Cañas et al. 2016; Palacio et al. in press).

Neighboring Ecuador and Venezuela also lack comprehensive taxonomic treatments for *Agrostis*: Ecuador has the aforementioned recent paper that describes *A.laegaardiana* and includes a brief key to similar species (Palacio et al. in press), checklists (Jørgensen and Ulloa-Ulloa 1994; Jørgensen and León-Yánez 1999) and Hitchcock’s (1927) synoptic treatment available; Venezuela has checklists (Hokche et al. 2008; Bono 2010) plus synoptic treatments for different regional areas (Briceño 2010; Dorr 2014). Thus, researchers have only these plus treatments from Central America (Pohl 1980; Pohl and Davidse 1994; Morales-Quirós 2003) and further south, such as those from Peru (Tovar 1993), Bolivia (Renvoize 1998) and Argentina (Rúgolo de Agrasar 2012), to help identify *Agrostis* taxa in Colombia.

The majority of *Agrostis* taxa cited for Colombia (Giraldo-Cañas et al. 2016; Palacio et al. in press) are found in high-elevation páramo grasslands. These ecosystems are located above the montane treeline of the humid tropical Andes from northern Peru to Colombia and Venezuela, with isolated regions also found in the mountains of Costa Rica and Panama (Luteyn 1999; Peyre et al. 2018). Páramos are classified as one of the world’s 34 biodiversity hotspots (Myers et al. 2000), with their high diversity and endemism probably a result of a unique geological and climatological history (Flantua et al. 2019). Colombia hosts the largest area of páramo of any country, holding 49% of the world’s páramo, which is an estimated c. 2 million hectares of national territory with the lower elevational limit ranging between 2850 and 3550 m alt. (Morales et al. 2007). Colombian páramos can be separated into five main high-elevation island-like enclaves within the country, namely the Cordillera Oriental, the Cordillera Central, the Cordillera Occidental, the Sierra Nevada de Santa Marta, and páramos of Nariño-Putumayo (Morales et al. 2007; Peyre et al. 2018). Roughly 60% of Colombia’s páramos are found in the Cordillera Oriental, with the political region (termed ‘Departamento’) Boyacá being especially important and hosting the largest area of páramo of any Departamento in Colombia, calculated at 19% of Colombia’s overall páramo (Morales et al. 2007). When conducting fieldwork in the páramos of Boyacá, *Agrostis* was found to be the most locally diverse grass genus (Sylvester pers. observation; unpubl. data), but with a notable dearth of taxonomic information available to help identify specimens. To support ecological and taxonomic research in Colombia’s páramos, we present a key to the species of *Agrostis* and *Podagrostis* currently (sometimes tentatively) accepted in Colombia, and short descriptions, images, and notes for the species found in páramos of Departamento Boyacá, Colombia.

## Materials and methods

Accepted species follow Soreng et al. (2003 and onwards). Herbarium acronyms follow Thiers (continuously updated). In this treatment, glabrous means without pubescence (in the sense of slender, relatively soft hairs unless otherwise stated). Smooth indicates no prickle-hairs with broad bases and/or hooked or pointed apices (i.e., pubescence can occur on a smooth surface, and a rough or scabrous surface can be glabrous). Taxonomic notes, including updated short descriptions mentioning the most important taxonomically informative characters for delineating species, are found for the eight species encountered during extensive fieldwork in the páramos of Boyacá. *Agrostissubrepens* (Hitchc.) Hitchc., which is mentioned in Giraldo-Cañas et al. (2016) as occurring in Departamento Boyacá, and *A.gigantea* Roth, *A.scabrifolia* Swallen and *A.lehmannii* Swallen, which are cited for Colombia in general (Giraldo-Cañas et al. 2016), are placed in the ‘Excluded species’ section at the end of the taxonomic treatment as no specimens were verified from Boyacá. *Agrostismeyenii* Trin., not registered for Colombia (Giraldo-Cañas et al. 2016), but which may occur there, is also included in the key. Notable synonyms of taxa found in Colombia or neighboring countries are included. The descriptions in the taxonomic notes were made based on specimens studied in both Colombia and neighboring countries, as well as information found in type protologues and literature (Pohl 1980; Tovar 1993; Pohl and Davidse 1994; Renvoize 1998; Morales-Quirós 2003; Briceño 2010; Rúgolo de Agrasar 2012; Dorr 2014). Only specimens studied from Departamento Boyacá are cited, or, exceptionally, specimens of interest from other Departamentos within Colombia are also cited. Specimen localities are cited by country (capital letters), political region (also historically called ‘departamento’; in bold) and then municipality. The herbaria COL, FMB, K, UPTC, and US were visited during the study. Only herbaria where specimens have been checked and verified by the authors have been cited.

## Taxonomic treatment

### Identification key to the 15 species of *Agrostis* and two species of *Podagrostis* that are cited in Colombia

(species that have not been confirmed by us to occur in Colombia are placed in brackets; *Agrostismeyenii*, which may occur in Colombia, but has so far not been registered, is also included)

**Table:** 

1	Palea absent or reduced, 0–1/3(–½) of the lemma in length	**2**
–	Palea well-developed, reaching from ½ to almost the full length of the lemma (rarely 2/5 the length of the lemma in *A.capillaris* and *A.stolonifera*)	**15**
2(1)	Panicle (at flowering) open, lax, usually ellipsoid, ovoid, obovoid, to pyramidal, not (sub-)spikelike, with upper lateral branches long and ascending or spreading but not held close to the central inflorescence axis, without spikelets close to the base (NB. Some species can have contracted panicles when immature or after flowering, but always diffuse at flowering)	**3**
–	Panicle (at flowering) moderately to densely congested, sub-spikelike or spikelike, with upper lateral branches short and held close to the central inflorescence axis, usually with spikelets present from the base or close to the base	**8**
3(2)	Lemma awned, awn (1.8−)2.5–4 mm long, flexuose to geniculate, twisted, inserted in the lower or middle 1/3 of the lemma	**4**
–	Lemma unawned or awn to 0.5 mm long and straight, not twisted, inserted in the middle or upper 1/3 of the lemma or subapical	**5**
4(3)	Culms scabrous, at least below the nodes; sheaths scaberulous; leaf blades rigid, scabrous on the abaxial and adaxial surfaces and margins	***A.scabrifolia* Swallen**
–	Culms smooth; sheaths smooth; leaf blades lax or less often semi-rigid (when involute), smooth throughout or finely scaberulous on the adaxial surface and/or margins	***A.mertensii* Trin.**
5(3)	Leaf blades convolute, involute, or conduplicate, rigid, 0.5–1 mm wide in diameter as rolled or folded; plants often stooling with notable pseudostolons and appearing long rhizomatous on herbarium sheets; culms slightly creeping or decumbent at their base but erect towards their apex; anthers 1−2 mm long	**6**
–	Leaf blades filiform, flat or conduplicate, lax, 0.2–6 mm wide, or rarely involute and firm in the basal leaves in *A.perennans* s.l.; plants usually without notable pseudostolons (sometimes apparent in *A.perennans* s.l.); culms usually erect, sometimes decumbent or stooling; anthers 0.7−1.1 mm long	**7**
6(5)	Culms 50−100 cm tall; panicles 5–10 cm wide; leaf blades with veins expressed and scabers pointed out right and left; flag ligules obtuse, 1−2.2 mm long; caryopsis 1−1.2 mm long; anthers 1−1.3 mm long	[***A.subrepens* (Hitchc.) Hitchc.**]
–	Culms (7−)24−60(−65) cm tall; panicles (0.5−)2−6 cm wide; leaf blades with veins not noticeably expressed (in Colombian material), usually smooth along the veins or lightly scaberulous with scabers in single file (in Colombian material); flag ligules acute to acuminate, (2.8−)3−6.7(−10) mm long; caryopsis 1.5−1.6 mm long; anthers 1−1.2(−2) mm long	**A.cf.imberbis Phil.**
7(5)	Leaf blades filiform, 0.2−1 mm wide, thin and flaccid; leaves mainly basal at maturity; panicles 4−12 cm × 2−6 cm; culms 10–40 cm tall	[***A.turrialbae* Mez**]
–	Leaf blades flat or conduplicate, (1–)1.5–3.5(–6) mm wide, sometimes involute in the basal leaves, usually thickened at the margins and keel, lax to slightly firm; leaves mainly basal early in the flowering season but tending to become mostly cauline with maturity; panicles often larger, (3.5−)10−22 × 2−11 cm; culms (21−)33−64(−100) cm tall	***A.perennans* (Walter) Tuck. s.l.**
8(2)	Lemma muticous, mucronate, or exceptionally with a short straight awn to ca. 1.3 mm long, subapical or inserted above the middle of the lemma, weak and falling easily, straight, not or barely exserted from the glumes	**9**
–	Lemma with a dorsal awn, (1.6–)2–6 mm long, persistant, twisted and bent, exerted from the glumes	**12**
9(8)	Leaf blades convolute, involute or strongly conduplicate (upper culm leaf blades sometimes flat, to 3 mm wide when opened out, in *A.boyacensis*), usually recurved, rigid, 0.25–1(–1.5) mm wide in diameter as folded or rolled; spikelets 1.5–2.5 mm long	**10**
–	Leaf blades filiform, flat, conduplicate or laxly rolled, straight or flexuous, lax and soft (sometimes involute, recurved and/or rigid in *A.tolucensis*), 1–5 mm wide when opened out; spikelets 2–4.1 mm long	**11**
10(9)	Tillers intravaginal, without cataphylls, not stooling and without notable lateral tending or ascending rhizomes; plants 3–12(–15) cm tall; spikelets 1.5–2.1 mm long (–2.5 mm in Bolivia?); all leaf blades similar, convolute or less often strongly conduplicate, recurved, rigid; widespread in páramos of Colombia	***A.breviculmis* Hitchc.**
–	Tillers extravaginal with cataphyllous shoots present, often with notable lateral tending or ascending rhizomes or stooling; plants 3–24 cm tall (plants from Sierra Nevada del Cocuy) or to 37 cm long (plants from elsewhere in Departamento Boyacá); spikelets 1.8–2.4(–2.5) mm long; leaf blades sometimes dimorphic, blades of tillers and lower culms convolute, involute, or sometimes strongly conduplicate, usually recurved or sometimes straight, firm to rigid, upper culm leaf blades sometimes flat, straight and firm; in Colombia, known from Departamento Boyacá, Cordillera Oriental	***A.boyacensis* Swallen & García-Barr.**
11(9)	Panicle branches, pedicels and central inflorescence axis usually smooth or exceptionally lightly scaberulous; culms 2–15(–30) cm tall, exceptionally taller; panicles 1–10 cm long, uninterrupted; spikelets 2.2–4.1 mm long; glumes subequal, keels usually scabrous in the distal 1/3, lower glume keel sometimes scabrous throughout or in the upper 2/3, upper glume keel sometimes completely smooth, glume surfaces smooth; lemma 1.7–2.6 mm long	[***A.meyenii* Trin.**]
–	Panicle branches, pedicels, and sometimes the central inflorescence axis moderately to densely scabrous; culms (3−)5.5−51(−80) cm tall, often > 15 cm tall; panicles (1−)2−15 cm long, often > 10 cm long, interrupted; spikelets 2−3(−3.5) mm long; glumes equal or subequal, keels and often surfaces scabrous at least in the distal half; lemma 1.4–2 mm long	***A.tolucensis* Kunth (= syn. *Agrostisglomerata* (J. Presl) Kunth)**
12(8)	Lemma with awn c. 6 mm long; ligules 7–8 mm long	***A.lehmannii* Swallen**
–	Lemma with awn (1.6–)2–3.5 mm long; ligules 1–8 mm long	**13**
13(12)	Leaf blades convolute, recurved, rigid, 0.5–2 mm wide when opened out; plants 3–12 cm tall; tillers intravaginal, plants without cataphyllous shoots or notable lateral tending or ascending rhizomes; ligules c. 1 mm long; glumes with coarse shiny hooks along the keel	***A.laegaardii* A.M. Molina & Rúgolo**
–	Leaf blades flat or folded, filiform or robust, straight, flexuous, or slightly recurved, lax to coriaceous and firm (sometimes convolute, recurved and rigid in *A.tolucensis* but then the culm blades are flat or folded), 1–6 mm wide when opened out; plants (3−)5.5−51(−80) cm tall; tillers extravaginal and intravaginal, plants usually with cataphyllous shoots and distinct lateral tending or short ascending rhizomes; ligules 2–8 mm long; glumes with minute or short hooks along the keel or smooth	**14**
14(13)	Leaf blades 2–6 mm wide, flat or folded, sometimes somewhat involute towards their apices, surfaces usually scabrous throughout, subcoriaceous to coriaceous; flag ligules 4–8 mm long; panicle 1–1.7(–2.5) cm wide, with primary lateral branches up to 7 cm long; spikelets 3–4.2 mm long; lemma 1.5–2 mm long, usually c. ½ the length of the glumes, rarely slightly longer	***A.foliata* Hook.f.**
–	Leaf blades 1–3(–5) mm wide, filiform, flat or folded, sometimes involute or convolute, scabrous in the margin and veins or smooth, lax to firm but not (sub-)coriaceous; flag ligules 2–4(−6.2) mm long; panicle 0.1–1.5 cm wide, with primary lateral branches 0.5–1.5 cm long; spikelets 2−3(−3.6) mm long; lemma 1.4–2 mm long, ½−2/3(−3/4) the length of the glumes	***A.tolucensis* Kunth**
15(1)	Leaf blades involute or convolute; panicles < 5 cm long; floret equalling or subequalling the glumes, usually with a short glabrous, smooth or scabrous rachilla emerging from under the palea (some spikelets within the inflorescence may lack the rachilla prolongation, so needs checking carefully); paleas reaching from (2/3) ¾ the length of the lemma to almost the apex of the lemma; lemmas muticous or with a short straight awn 0.2–0.5 mm long, inserted medially or in the upper half of the lemma, not surpassing the glumes	**16**
–	Leaf blades generally flat (*A.capillaris* often with basal blades involute and culm blades flat); panicles generally > 5 cm long (sometimes to 2 cm long in *A.stolonifera* or 3 cm in *A.capillaris*); floret notably shorter than the glumes, usually 1/3–3/4 the length of the glumes, without a rachilla prolongation; paleas reaching from (2/5–)2/3–3/4 the length of the lemma; lemmas muticous or with an awn of varying length, ranging from a short straight awn, 0.2–1 mm long, to a long geniculate and twisted awn to 4 mm long, inserted basally, medially or in the upper half of the lemma, not surpassing or greatly surpassing the glumes	**17**
16(15)	Panicles contracted and slender, c. 0.5 cm wide, spikelets present from near the base; spikelets 2.2–4.2 mm long; upper glume 3-veined; plants forming small tussocks; anthers 1.5–2.2 mm long	***Podagrostiscolombiana* Sylvester & Soreng**
–	Panicles lax and open, 1–2(–3) cm wide, spikelets in the distal 1/3, the lower 2/3 naked; spikelets 1–1.5 mm long; upper glume 1-veined; plants forming short tufts; anthers 0.4–1 mm long	***Podagrostistrichodes* (Kunth) Sylvester & Soreng**
17(15)	Ligules of basal leaves and tillers ≤ 1 mm long, distinctly shorter than wide; ligules of culm leaves 0.5–1.5(–2.9) mm long, shorter to sometimes longer than wide	***A.capillaris* L.**
–	Ligules of basal leaves and tillers 1–3 mm long, as long as or distinctly longer than wide; ligules of culm leaves 2–8 mm long, as long as or distinctly longer than wide	**18**
18(17)	Plants erect or decumbent, usually without stolons but always with extensively creeping rhizomes; panicle lax and open after flowering, lateral branches extended at maturity, spikelets absent in the proximal half; palea (0.8–)1.2–1.6 mm long	***A.gigantea* Roth**
–	Plants generally creeping, usually extensively stoloniferous, rhizomes usually absent; panicle tightly contracted after flowering, lateral branches held close to the central rachis at maturity, spikelets present in the proximal half; palea 0.7–1.3(–1.6) mm long	***A.stolonifera* L.**

### Clave de identificación para las 15 especies de *Agrostis* y dos especies de *Podagrostis* citadas para Colombia

(las especies que no han sido confirmadas por nosotros que ocurran en Colombia se indican entre paréntesis; *Agrostismeyenii*, la cual puede ocurrir en Colombia, pero que hasta ahora no ha sido registrada, es también incluida).

**Table:** 

1	Pálea ausente o reducida, 0–1/3(–½) de la longitud de la lema	**2**
–	Pálea bien desarrollada, que alcanza desde ½ a casi toda la longitud de la lema (rara vez 2/5 de la longitud de la lema en *A.capillaris* y *A.stolonifera*)	**15**
2(1)	Panícula (durante la floración) abierta, laxa, generalmente elipsoide, ovoide, obovoide, a piramidal, no (sub-) espiciforme, con ramificaciones laterales superiores largas y ascendentes o extendidas, pero no próximas al eje central de la inflorescencia, sin espiguillas cerca de la base (NB. Algunas especies pueden presentar las panículas contraídas cuando inmaduras y después de la floración, pero siempre abiertas en la floración)	**3**
–	Panícula (durante la floración) de moderada a densamente congesta, sub-espiciforme a espiciforme, con ramificaciones laterales superiores cortas y próximas al eje central de la inflorescencia, usualmente con espiguillas presentes desde la base o cerca de la base	**8**
3(2)	Lema aristada, arista (1.8–)2.5–4 mm de largo, flexuosa a geniculada, retorcida, insertada 1/3 en la parte inferior o media de la lema	**4**
–	Lema no aristada o con arista 0.5 mm de largo y recta, no retorcida, insertada 1/3 en la parte media o superior de la lema o subapical	**5**
4(3)	Culmos escabrosos, al menos debajo de los nudos; vainas escabriúsculas; láminas foliares rígidas, escabrosas en la superficie abaxial y adaxial y los márgenes	***A.scabrifolia* Swallen**
–	Culmos lisos; vainas lisas; láminas foliares laxas, o menos frecuente semirigidas (cuando involutas), lisas o finamente escabriúsculas a lo largo de la superficie adaxial y/o los márgenes	***A.mertensii* Trin.**
5(3)	Láminas foliares convolutas, involutas, o conduplicadas, rígidas, 0.5–1 mm de diámetro cuando enrolladas o dobladas; hierbas estoloníferas provistas de notables pseudo-estolones que aparentan largos rizomas en los especímenes de herbario; culmos ligeramente rastreros o decumbentes en su base, pero erectos hacia su ápice; anteras 1–2 mm de largo	**6**
–	Láminas foliares filiformes, planas o conduplicadas, laxas, 0.2–6 mm de ancho, o raramente involutas y firmes en las hojas basales de *A.perennans* s.l.; plantas usualmente sin pseudoestolones notables (a veces evidentes en *A.perennans* s.l.); culmos generalmente erectos, a veces decumbentes o estoloníferos; anteras 0.7–1.1 mm de largo	**7**
6(5)	Culmos 50–100 cm de alto; panículas 5–10 cm de ancho; láminas foliares con costillas conspicuas y espínulas señalando a derecha e izquierda; lígulas bandera (i.e., lígula de la última hoja de la caña florífera) obtusas, 1–2.2 mm de largo; cariopsis 1–1.2 mm de largo; anteras 1–1.3 mm de largo	[***A.subrepens* (Hitchc.) Hitchc.**]
–	Culmos (7−) 24−60 (−65) cm de alto; panículas (0.5−) 2−6 cm de ancho; láminas foliares con venas no expresadas notablemente (en material Colombiano), generalmente lisas a lo largo de las venas o ligeramente escabriúsculas con espínulas dispuestas en un sola fila (en material Colombiano); lígulas bandera agudas a acuminadas, (2.8−)3−6.7(−10) mm de largo; cariopsis 1.5−1.6 mm de largo; anteras 1−1.2 (−2) mm de largo	**A.cf.imberbis Phil.**
7(5)	Láminas foliares filiformes, 0.2−1 mm de ancho, delgadas y flácidas, hojas basales maduras; panículas 4−12 × 2−6 cm; culmos 10−40 cm de altura	[***A.turrialbae* Mez**]
–	Láminas foliares planas o conduplicadas, (1–)1.5–3.5(–6) mm de ancho, a veces involutas en las hojas basales, generalmente engrosadas en los márgenes y en la quilla, laxas a ligeramente firmes; hojas en su mayoría basales a principios de la floración y que tienden a convertirse en caulinares en la madurez; panículas a menudo más grandes, (3.5−)10−22 × 2−11 cm; culmos hasta (21−)33−64(−100) cm de alto	***A.perennans* (Walter) Tuck. s.l.**
8(2)	Lema mútica, mucronada, o excepcionalmente con una arista corta y recta hasta 1.3 mm de largo, subapical o inserta por encima de la mitad de la lema, débil y fácilmente desarticulable, recta, no o escasamente exerta de las glumas	**9**
–	Lema con arista dorsal, (1.6–)2–6 mm de largo, persistente, retorcida y doblada, exerta de las glumas	**12**
9(8)	Láminas foliares convolutas, involutas, o fuertemente conduplicadas (láminas foliares del culmo superior a veces planas, de 3 mm de ancho cuando abiertas, en *A.boyacensis*), usualmente recurvadas, rígidas, 0.25–1(–1.5) mm de diámetro tanto plegadas como enrolladas; espiguillas 1.5–2.5 mm de largo	**10**
–	Láminas foliares filiformes, planas, conduplicadas o laxamente enrolladas, rectas o flexibles, laxas y blandas (a veces involutas, recurvadas y/o rígidas en *A.tolucensis*), 1–5 mm de ancho cuando extendidas; espiguillas 2–4.1 mm de largo	**11**
10(9)	Innovaciones intravaginales, sin catáfilos, no reptante y sin notables rizomas tendidos lateralmente o ascendentes; hierbas 3–12(–15) cm de alto; espiguillas 1.5–2.1 mm de largo (–2.5 mm en especímenes originarios de Bolivia?); todas las láminas foliares son similares, convolutas o con menos frecuencia fuertemente conduplicadas, recurvadas, rígidas; ampliamente distribuidas en los páramos de Colombia	***A.breviculmis* Hitchc.**
–	Innovaciones extravaginales, catáfilos con vástagos axilares presentes, a menudo con rizomas laterales inclinados o ascendentes notables, o reptantes; hierbas 3–24 cm de alto (plantas de la Sierra Nevada del Cocuy) o de 37 cm de largo (plantas de otras partes del Departamento de Boyacá); espiguillas 1.8–2.4(–2.5) mm de largo; láminas foliares a veces dimórficos, láminas de las innovaciones y hojas caulinares inferiores convolutas, involutas o a veces fuertemente conduplicadas, generalmente recurvadas o algunas veces rectas, firmes a rígidas; láminas de las hojas caulinares superiores algunas veces planas, rectas y firmes; nativas en Colombia, presencia confirmada en el Departamento de Boyacá, Cordillera Oriental	***A.boyacensis* Swallen & García-Barr.**
11(9)	Panículas con ramas, pedicelos y eje central de la inflorescencia generalmente lisos o por excepción ligeramente escabriúsculos; culmos 2–15 (–30) cm de alto, excepcionalmente más altos; panículas 1–10 cm de largo, ininterrumpidas; espiguillas 2.2–4.1 mm de largo; glumas subiguales, quillas usualmente escabrosas en el 1/3 distal, a lo largo de la quilla de la gluma inferior generalmente escabrosa o 2/3 de la parte superior, quilla de la gluma superior a veces completamente lisa, superficies de las glumas lisas; lema 1.7–2.6 mm de largo	[***A.meyenii* Trin.**]
–	Panículas con ramas, pedicelos y, a veces, el eje central de la inflorescencia de moderado a densamente escabroso; culmos (3−)5.5−51(−80) cm de alto, a menudo > 15 cm de alto; panículas (1−)2−15 cm de largo, a menudo > 10 cm de largo, interrumpidas; espiguillas 2−3(−3.5) mm de largo; glumas iguales o subiguales, quillas y a menudo la superficie escabrosas al menos en la mitad distal; lema 1.4–2 mm de largo	***A.tolucensis* Kunth (= syn. *Agrostisglomerata* (J. Presl) Kunth)**
12(8)	Lema con arista c. 6 mm de largo; lígulas 7–8 mm largo	***A.lehmannii* Swallen**
–	Lema con arista (1.6–)2–3.5 mm de largo; lígulas 1–8 mm largo	**13**
13(12)	Láminas foliares convolutas, recurvadas, rígidas, de 0.5–2 mm de ancho cuando están abiertas; hierbas 3–12 cm de alto; innovaciones intravaginales, plantas sin catáfilos ni vástagos axilares ni notables rizomas laterales ni ascendentes; lígulas c.1 mm de largo; glumas con espinas gruesas y brillantes a lo largo de la quilla	***A.laegaardii* A.M. Molina & Rúgolo**
–	Láminas foliares planas o dobladas, filiformes o robustas, rectas, flexuosas o ligeramente recurvadas, laxas a coriáceas y firmes (generalmente convolutas, recurvadas y rígidas en *A.tolucensis* pero luego las láminas del culmo son planas o plegadas), 1–6 mm de ancho al extenderse; hierbas (3−)5.5−51(−80) cm de alto; innovaciones extravaginales e intravaginales, usualmente catáfilos con vástagos axilares y evidentes rizomas laterales o corto ascendentes; lígulas 2–8 mm de largo; glumas con espinas diminutas o cortas a lo largo de la quilla o lisas	**14**
14(13)	Láminas foliares 2–6 mm de ancho, planas o a veces algo involutas hacia el ápice, superficies usualmente escabrosas en todo su largo, subcoriáceas a coriáceas; lígulas bandera 4–8 mm de largo; panícula 1–1.7(–2.5) cm de ancho, con ramas primarias laterales de hasta 7 cm de largo; espiguillas 3–4.2 mm de largo; lema 1.5–2 mm de largo, usualmente c. ½ la longitud de las glumas, rara vez un poco más larga	***A.foliata* Hook.f.**
–	Láminas foliares 1–3 (–5) mm de ancho, planas o dobladas, a veces involutas o convolutas, escabrosas en el margen y en las venas o lisas, laxas a firmes, pero no (sub-) coriáceas; lígulas bandera 2–4(–6.2) mm de largo; panícula 0.1–1.5 cm de ancho, con ramas primarias laterales 0.5–1.5 cm de largo; espiguillas 2–3(–3.6) mm de largo; lema 1.4–2 mm de largo, c. ½−2/3(−3/4) la longitud de las glumas	***A.tolucensis* Kunth**
15(1)	Láminas foliares involutas o convolutas; panículas < 5 cm de largo; antecio que iguala o sub-iguala a las glumas, usualmente con una raquilla corta, glabra, lisa o escabrosa que emerge por debajo de la pálea (algunas espiguillas dentro de la inflorescencia pueden carecer de la prolongación de la raquilla, por ello se necesita revisar cuidadosamente); paleas que alcanzan desde (2/3) ¾ la longitud de la lema a casi el ápice de la lema; lemas múticas o con una arista corta y recta 0.2–0.5 mm de largo, insertada medialmente o en la mitad superior de la lema, sin sobrepasar las glumas	**16**
–	Láminas foliares generalmente planas (*A.capillaris* a menudo con láminas basales involutas y las láminas caulinares planas); panículas generalmente > 5 cm de largo (algunas veces hasta 2 cm de largo en *A.stolonifera* o 3 cm en *A.capillaris*); antecio notablemente más corto que las glumas, usualmente 1/3–3/4 de la longitud de las glumas, sin una prolongación de la raquilla; paleas que alcanzan desde (2/5–)2/3–3/4 la longitud de la lema; lemas múticas o con una arista de longitud variable, que van desde una arista corta y recta de 0.2–1 mm de largo, a una arista larga, geniculada y retorcida de 4 mm de largo, insertada basalmente, hacia la mitad o en la mitad superior de la lema, sin sobrepasar o superar en gran medida las glumas	**17**
16(15)	Panículas contraídas y delgadas, c. 0.5 cm de ancho, con espiguillas desde cerca de la base; espiguillas 2.2–4.2 mm de largo; gluma superior 3-nervada; plantas que forman pequeños macollos; anteras 1.5–2.2 mm de largo	***Podagrostiscolombiana* Sylvester & Soreng**
–	Panículas laxas y abiertas 1–2(–3) cm en ancho, espiculadas en el 1/3 distal, los 2/3 inferiores desnudas; espiguillas 1–1.5 mm de largo; gluma superior 1-nervada; plantas que forman pequeños mechones; anteras 0.4–1 mm de largo	***Podagrostistrichodes* (Kunth) Sylvester & Soreng**
17(15)	Lígulas de las hojas basales e innovaciones ≤ 1 mm de largo, claramente más cortas que anchas; lígulas de las hojas caulinares 0.5–1.5(–2.9) mm de largo, más cortas hasta a veces más largas que anchas	***A.capillaris* L.**
–	Lígulas de las hojas basales e innovaciones 1–3 mm de largo, tan largas o claramente más largas que anchas; lígulas de las hojas caulinares 2–8 mm de largo, tan largas o claramente más largas que anchas	**18**
18(17)	Plantas erectas o decumbentes, usualmente sin estolones, pero siempre con rizomas extensivamente rastreros; panícula laxa y abierta después de la floración, ramas laterales extendidas en la madurez, espiguillas ausentes en la mitad proximal; pálea (0.8–)1.2–1.6 mm de largo	***A.gigantea* Roth**
–	Plantas generalmente rastreras, usualmente extensivamente estoloníferas, rizomas usualmente ausentes; panícula estrechamente contraída después de la floración, ramas laterales próximas al raquis central en la madurez, espiguillas presentes en la mitad proximal; pálea 0.7–1.3(–1.6) mm de largo	***A.stolonifera* L.**

### Taxonomic notes for the species of Agrostis and Podagrostis found in páramos of Departamento Boyacá, Colombia

#### Agrostis

Taxon classificationPlantaePoalesPoaceae

L. Sp. Pl. 1: 61. 1753

16EBC6BF-A257-5990-9B05-29CDFEA38C7C

= Vilfa Adans., Fam. Pl. 2: 495. 1763. Type: Vilfastolonifera (L.) P. Beauv. (lectotype, designated by Hitchcock 1920: 127).
= Trichodium Michx., Fl. Bor.-Amer. (Michaux) 1: 41. 1803. Type: Trichodiumlaxiflorum Michx. (lectotype, designated by Hitchcock 1920: 127). Many other heterotypic synonyms.

##### Type.

*Agrostisstolonifera* L. (lectotype, designated by Hitchcock 1920: 125)

##### Description.

**Annuals or perennials. Leaves** basal or cauline; **ligules** membranous to scarious. **Inflorescence** a panicle, lax and open to contracted and spikelike. **Spikelets** 1-flowered, disarticulating above the glumes, laterally compressed; **glumes** as long as the spikelet, equal or subequal, persisting on the plant after the florets have fallen, usually 1-veined, rarely 3-veined; **floret** usually notably shorter than the glumes or reaching to ¾ the length of the glumes, exceptionally longer; **lemmas** membranaceous or hyaline, generally thinner than the glumes, dorsally rounded, 3- or 5-veined, veins not or distinctly evident; **paleas** often absent or noticeably shorter than the lemma, sometimes reaching to ¾ the length of the lemma, hyaline and slightly to notably thinner than the lemmas, keels usually obscure, rarely distinct, glabrous, usually smooth, rarely scaberulous; **calluses** rounded, glabrous or pubescent and usually with 2 lateral tufts of short hairs; **rachilla** prolongation absent. **Flowers** perfect; **anthers** 3 in number, 0.3–1.8 mm long. **Caryopses** hard (in species from Colombia) or sometimes with liquid endosperm.

##### Notes.

In Colombian páramos, taxa of *Agrostis* can be most easily confused with those of *Calamagrostis* s.l. (i.e., *Cinnagrostis* Griseb., *Deschampsia* P. Beauv., *Paramochloa* P.M. Peterson, Soreng, Romasch. & Barberá, *Peyritschia* E. Fourn.; Peterson et al. 2019; Sylvester et al. 2019a), *Podagrostis*, *Polypogon* Desf., and *Sporobolus* R. Br. The genera previously circumscribed as *Calamagrostis* s.l. (Peterson et al. 2019; Sylvester et al. 2019a) can usually be differentiated by a combination of a prolonged hairy rachilla emerging from the base of the floret, a well-developed palea, a hairy callus, an awn present and inserted dorsally on the lemma, and an upper glume with well-developed lateral veins, although certain species are missing some of these characteristics (see Sylvester et al. 2019a). *Polypogon* is principally differentiated by spikelets that disarticulate below the glumes, with the grain, lemma, palea, glumes and part of the pedicel falling together. The glumes are also often awned in *Polypogon. Sporobolus* is principally differentiated by its ligule in the form of a line of hairs, its well-developed paleas with the same consistency as the lemma, and the lemmas being 1(–3) veined.

#### Agrostis boyacensis

Taxon classificationPlantaePoalesPoaceae

Swallen & García-Barr., Caldasia 2 (8): 303, fig. A. 1943

043971C1-07AC-5C2B-9BAE-A85415594868

Figs 1 , 2

##### Type.

Colombia. Boyacá: Nevado del Cocuy, alto valle de Las Lagunillas, [6.3906N, 72.3542W], 4000–4300 m alt., 12 Sep. 1938, J. Cuatrecasas & H. García-Barriga 1459 (holotype: US (US00131729 [image!]); isotype: COL (COL000006092!), SI (SI000494 [image!] fragm. ex US)).

##### Description.

**Perennial herbs**, densely tufted, often stooling with perenniating many-branched culms, often with short lateral tending to ascending rhizomes. **Tillers** extravaginal, with cataphyllous shoots present. **Culms** 3−24 cm tall (to 37 cm tall in specimens from Boyacán páramos outside the Sierra Nevada del Cocuy), erect to arched, firm, with 0(−2) nodes exerted at flowering, densely scabrous throughout or scabrous just below the nodes, or rarely smooth (specimens from Boyacán páramos outside the Sierra Nevada del Cocuy). **Leaves** basal and cauline, often mainly basal, glabrous, usually densely scabrous throughout, less often smooth or scaberulous (specimens from Boyacán páramos outside the Sierra Nevada del Cocuy); **ligules** 0.9−2 mm long, membranous or slightly scarious, truncate to triangular and obtuse, moderately to strongly decurrent with the sheath, abaxial surface smooth or scaberulous; **blades** (2−)3−6(−12) cm long, 0.5−1.2(−1.5) mm in diameter as folded or rolled, convolute, involute, or strongly conduplicate, usually recurved or sometimes straight, firm to rigid, those of upper culm sometimes flat, firm, 2−3 mm wide, abaxial surface usually densely scabrous throughout, less often smooth to scaberulous (specimens from Boyacán páramos outside the Sierra Nevada del Cocuy), adaxial surface and margins moderately to densely scabrous, apices blunt or slightly naviculate-acute. **Panicles** (2−)3−6(−8) cm long, c. 0.3−0.7(−0.9) cm wide, moderately to densely congested, sub-spikelike to spikelike, generally not interrupted, subincluded in the basal foliage to greatly exerted, lateral branches with spikelets usually present almost to the base, upper lateral branches short and held close to the central inflorescence axis, central axis and panicle branches moderately to densely scabrous, rarely smooth or scaberulous (specimens from Boyacán páramos outside the Sierra Nevada del Cocuy); **pedicels** 0.8−2.5 mm long, usually shorter than their spikelets, not obviously dilated at their apex, moderately to densely scabrous, sometimes smooth or scaberulous (specimens from Boyacán páramos outside the Sierra Nevada del Cocuy). **Spikelets** 1.8−2.4(−2.5) mm long; **glumes** usually unequal, sometimes subequal, the lower longer than the upper by up to 0.3 mm and usually wider than the upper, 1-veined, lower glume keel scabrous throughout to only in distal 1/3, upper glume scabrous only in the distal 1/3 or sometimes smooth throughout, apices acute; **floret** 3/5−2/3(−3/4) the length of the glumes; **calluses** glabrous or with 2 sparse tufts of very short hairs on the lateral sides; **lemmas** 1−1.5 mm long, glabrous, smooth, 5-veined, apex obtuse to slightly truncate, erose, muticous or exceptionally with a short straight awn inserted in the upper ½ of the lemma, to 0.5 mm long, not surpassing the glume apex, weak and falling easily (i.e. Sylvester 3071); **paleas** absent or to 0.3 mm long, < ¼ the length of the lemma; **rachilla** absent; **anthers** 0.5−0.8(−1) mm long.

**Figure 1.**
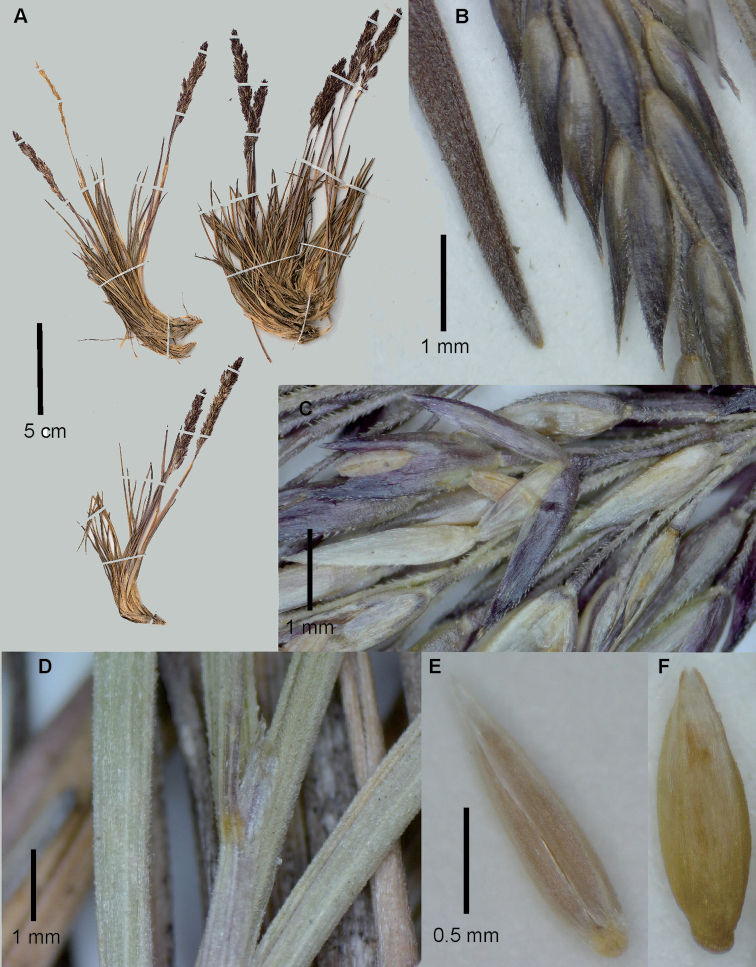
*Agrostisboyacensis*, examples of specimens from páramo or superpáramo of the Sierra Nevada del Cocuy **A** whole plant **B** close-up of inflorescence **C** spikelet at anthesis, lateral view **D** upper culm blades and ligular area **E** floret, ventral view **F** floret, dorsal view. Images **A, C, D, F**: Cleef 8504 (US01247250) courtesy of United States National Herbarium (US); **B, E**: Cleef 5665 (US2785695). Scale bar of **E** also for **F**.

##### Distribution and ecology.

Colombia, Ecuador?. Páramos and superpáramos, found in both zonal grass páramo and azonal high elevation moraine, 2850–4500 m alt. Within Colombia, the species seems to be found only in the Cordillera Oriental, with specimens only seen from Departamento Boyacá. Luteyn (1999) mentions the species to be found in Ecuador and the species is also mentioned in the key to some Ecuadorian taxa (Palacio et al. in press), but no specimens have been verified at US.

##### Other specimens examined.

**Colombia**. **Boyacá**: Munic. Chiscas, Páramo de Chacaritas, found on a rock escarpment 4 m tall, 6.62227N, 72.39040W, 4192 m alt., 4 Mar. 2018, S.P. Sylvester 3117 (K, UPTC, US); Munic. Chiscas, Páramo de Chacaritas, close to the foot of the talus scree slope, 6.61779N, 72.38899W, 4354 m alt., 4 Mar. 2018, S.P. Sylvester 3129 (COL, FMB, K, US); Sierra Nevada del Cocuy, Laguna Grande, in open gravelly slopes, morranes and similar places at high altitude, [6.5556N, 72.3253W], 4300−4500 m alt., 28 Dec. 1985, J.R.I. Wood 5247 (US3481052); Munic. Duitama, Páramo de la Rusia, Sector del Páramo de Agueros, en la vía que conduce a la vereda Avendaños, páramo dominado por *Chusquea*, evidencia de pastoreo intenso, en alguna perturbación humano / animal, 5.94611N, 73.08481W, 3768 m alt., 3 Oct. 2017, S.P. Sylvester 3017a (COL, K, SI, US); Munic. Arcabuco, Vereda el Carmen, Páramo del Valle, páramo muy húmedo dominado por el grupo *Chusquea*, pastoreo natural, 5.75425N, 73.38303W, 3430 m alt., 15 Nov. 2017, S.P. Sylvester 3071 (K, US, SI). Munic. Chiscas, Páramo de Chacaritas, rocas expuestas en vegetación de páramo, 6.61931N, 72.38898W, 4287 m alt., 4 Mar. 2018, S.P. Sylvester 3124 (K, US, SI). Munic. Mongua, Páramo de Ocetá, valle de Laguna Negra, vegetación de frailejonal pajonal, con presencia de arbustos pequeños, Se observan rastros de pastoreo, 5.69525N, 72.79133W, 3694 m alt., 29 Nov. 2017, L.E. Cuta-Alarcón 353c (US); Planos del Toldadero, [6.3669N, 72.3342W], 3950 m alt., 13 Sep. 1938, J. Cuatrecasas 1554 (US17730370); Sierra Nevada del Cocuy, alrededores de Salto de Correlitos, [6.4444N, 72.3175W], 4400 m alt., 14 Apr. 1959, H.G. Barclay 7394 (US2434358); Sierra Nevada del Cocuy, Alto Ritacuva, [6.5139N, 72.3531W], 4450 m alt., between wet slopes which have dense *Espeletia*, 24 Apr. 1959, H.G. Barclay 7453 (US2434361); Sierra Nevada del Cocuy, alrededores de Salto de Correlitos, rocky, southwest-facing slope, better vegetated between rocky ridges, east of Laguna San Paulino, [6.4444N, 72.3175W], 4300 m alt., 14 Apr. 1959, H.G. Barclay 7381 (US2434355); Sierra Nevada del Cocuy, alto valle de Las Lagunillas, [6.3469N, 72.3261W], 4000−4300 m alt., 12 Sep. 1938, J. Cuatrecasas 1465 (US1772998); Sierra Nevada del Cocuy, Alto Valle Lagunillas, morrena seca pedregosa 100 m al SE de la Laguna Cuadrada, [6.3619N, 72.3344W], 4080 m alt., con *Calamagrostiseffusa* [(Kunth) Steud. = *Paramochloaeffusa* (Kunth) P.M. Peterson, Soreng, Romasch. & Barberá] y Gymnomitriaceae predominantes, asociadas con *Luzula*, *Espeletiacolombiana* [Cuatrec.] y musgos, 26 Nov. 1972, A.M. Cleef 5535 (US2785729); Sierra Nevada del Cocuy, Alto Valle Lagunillas, [6.3906N, 72.3542W], 3995 m alt., con *Espeletiacolombiana* predominante, asociada con *Aciachnepulvinata* [Benth.], *Agrostisbreviculmis*, *Agrostistrichodes* [*Podagrostistrichodes*] y *Acaenacylindristachya* [Ruiz & Pav.], gramínea común, 25 Sep. 1972, A.M. Cleef 5504 (US2785744); Sierra Nevada del Cocuy, Boqueron de Cusiri, límite superpáramo y páramo propiamente dicho, vertiente seco muy pedregoso, [6.3431N, 72.3128W], 4320 m alt., con *Calamagrostiseffusa* [=*Paramochloaeffusa*] y *Espeletiacleefii* [Cuatrec.], gramínea común, 5 Mar. 1973, A.M. Cleef 8796 (US2785680); Sierra Nevada del Cocuy, Laguna Grande de la Sierra, [6.5556N, 72.3253W], 4300−4500 m alt., 28 Dec. 1985, J.R.I. Wood 5247 (US3481052); Sierra Nevada del Cocuy, Páramo Cóncavo, Cuchilla Puentepiedra casi 2km al NE de la Laguna Pintada, vertiente pedregoso seco, [6.3723N, 72.3175W], 4510 m alt., con *Calamagrostiseffusa* [=*Paramochloaeffusa*], *Poa* sp., asociadas con *Espeletialopezii* fma. [Cuatrec.], y musgos, 30 Sep. 1972, A.M. Cleef 5665 (US2785695); Sierra Nevada del Cocuy, Páramo Cóncavo, morrena seca en el límite páramo propiamente dicho y superpáramo, 3.5 km al NNW del Morro Pulpito del Diablo, [6.3995N, 72.3123W], 4325m alt., matorral de *Seneciovaccinioides* [Cuatrec.] con *Alchemilla* sp., 1 Mar. 1973, A.M. Cleef 8680 (US2785780); Sierra Nevada del Cocuy, Páramo Cóncavo, superpáramo, morrena seca 3 km aprox. al Norte del morro Pulpito del Diablo. [6.3995N, 72.3123W], 4375m alt., rastrojo de *Senecioguicanensis* [Cuatrec.], gramínea común, 28 Feb. 1973, A.M. Cleef 8627 (US2785676); Sierra Nevada del Cocuy, Páramo Cóncavo, superpáramo 3.5 km aprox. al NNW del morro Pulpito del Diablo, morrena seca muy pedegrosa, [6.3995N, 72.3122W], 4315 m alt., con *Luzula* y Pernettyaprostratavar.prostrata [(Cav.) DC.], gramínea común, 26 Feb. 1973, A.M. Cleef 8504 (US01247250); Sierra Nevada del Cocuy, Páramo Cóncavo, superpáramo abrigo rocoso denominado Cueva de los Hombres, 3 km aprox. al N del morro Pulpito del Diablo, [6.3995N, 72.3122W], 4350 m alt., 28 Feb. 1973, A.M. Cleef 8608 (US2785679); Sierra Nevada del Cocuy, Quebrada Bocatoma, vertiente N del valle, 800 m al ENE [East North-East] de la Laguna Pintada, [6.4795N, 72.3167W], 4060 m alt., *Stephaniella* sp. predominante asociada con *Calamagrostiseffusa* [=*Paramochloaeffusa*], *Alchemillapolylepis* [Wedd.], *Achyroclinelehmanii* [Hieron.] y *Polytrichum* sp., 29 Sep. 1972, A.M. Cleef 5648 (US2785702).

##### Notes.

**Figure 2.**
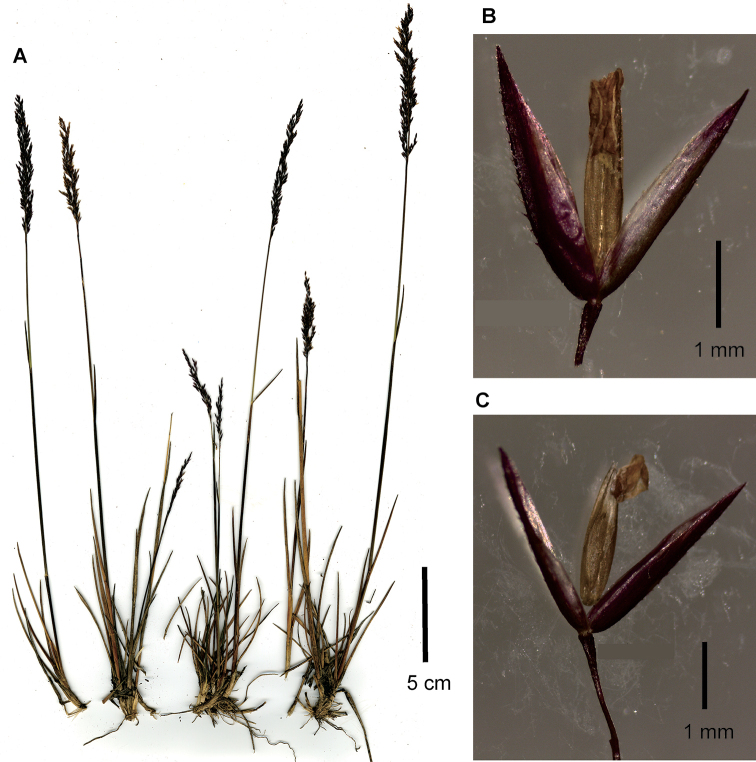
*Agrostisboyacensis*, example of a specimen encountered in páramos of Boyacá outside of the Sierra Nevada del Cocuy **A** whole plant **B** spikelet, glumes lateral view and floret ventral view showing anthers **C** spikelet, glumes lateral view with floret detached and raised above the glumes showing ventral surface. Images of Sylvester 3017a (FMB).

Specimens encountered from páramos not belonging to the Sierra Nevada del Cocuy, but within Departamento Boyacá, bear notable differences from those from the type locality and further study is needed to elucidate whether these are a distinct species or subspecies of *A.boyacensis*. These differences include leaf sheaths and blades, panicle branches, and pedicels being usually smooth or very lightly scaberulous, and plants being larger, usually > 20 cm tall and up to 37 cm tall, and with the panicles largely exerted from the basal foliage (Fig. 2). These characteristics place it close to *A.meyenii*, which is not known from Colombia (Giraldo-Cañas et al. 2016) or páramos in general (Luteyn 1999), although it is mentioned in the recently-published abbreviated key to some Ecuadorian species (Palacio et al. in press) (see below for how to differentiate these species).

The habit of type specimens and some of the other specimens examined (e.g. Sylvester 3117 and 3129), with perenniating, many-branched culms, appears to be related to the habitat, with the Sylvester 3117 and 3129 specimens being found growing amongst moss. The leaf blades of these were also slightly laxer, albeit still involute or strongly conduplicate. In harsher conditions, such as open gravelly slopes, the tufts are more compact and leaf blades are rigid and strongly rolled and resemble a very large, densely-tufted *A.breviculmis* with slightly laxer panicles.

##### Similar species.

*Agrostisbreviculmis* and *A.meyenii* both have congested spikelike panicles and florets that lack prominent awns and well-developed paleas. *Agrostisbreviculmis* bears close similarity in its convolute, often recurved, rigid to firm leaf blades that usually measure < 1 mm wide in diameter, and small spikelets with fairly well developed coarse scabers on the glume keels. *Agrostisboyacensis* can be distinguished from *A.breviculmis* principally by the habit, with many extravaginally branched culms that form large dense tufts to 37 cm tall (vs. intravaginal innovations forming short tufts to 12(−15) cm tall in *A.breviculmis*), laxer panicles, 3–9 mm wide (vs. c. 0.5−2 mm wide in *A.breviculmis*), and slightly larger spikelets, 1.8–2.4(–2.5) mm long (vs. usually 1.5–2.1 mm long in *A.breviculmis*, noted to reach 2.5 mm long in Bolivia [Renvoize 1998]).

*Agrostisboyacensis* can be distinguished from *A.meyenii* principally by its robust convolute, involute or strongly conduplicate, usually recurved and rigid leaf blades (vs. laxer, weaker, flat or folded, usually filiform leaf blades in *A.meyenii*). All specimens encountered from the Sierra Nevada del Cocuy can be easily differentiated by their panicle branches, pedicels, and leaf sheaths and blades that are moderately to densely scabrous (vs. panicle branches and pedicels smooth to lightly scaberulous, leaf sheaths smooth, blades smooth or scabrous in *A.meyenii*). Specimens collected in other páramos from Departamento Boyacá, outside of the Sierra Nevada del Cocuy, bear further similarities with *A.meyenii*, such as their culms and inflorescences being longer and greatly exerted from the basal foliage, and panicle branches, pedicels, and leaf blades that are usually smooth to lightly scaberulous (Fig. 2). Nevertheless, these can be differentiated from *A.meyenii* by a) their robust convolute, involute or strongly conduplicate, rigid and usually recurved leaf blades; b) the glumes usually being unequal, with the lower glume longer and usually wider than the upper; and c) the lower glume keel scabrous throughout to only in distal 1/3, the upper glume keel scabrous only in the distal 1/3 or sometimes smooth throughout.

*Agrostistolucensis* has a congested spikelike panicle and florets with a minute palea and a lemma that can sometimes lack awns (see notes under *A.tolucensis*). This species usually has leaf blades filiform, flat, or folded, lax to firm, but can sometimes have basal leaf blades involute or convolute and firm to rigid. All specimens examined at US with involute or convolute and rigid leaf blades had lemmas with a well-developed dorsally inserted awn. Further distinction from *A.boyacensis* can be found in how the leaf blades are smooth or scabrous only on the margin and sometimes veins in *A.tolucensis*, while scabrous throughout (margin, veins, and in-between veins) in *A.boyacensis* from the Sierra Nevada del Cocuy, although *A.boyacensis* specimens from other Boyacan páramos have blades smooth to scaberulous.

The sometimes strongly conduplicate leaf blades of this species can also give it a resemblance to *A.foliata*, which has subcoriaceous to coriaceous leaf blades that can sometimes be folded, although these are usually > 1.5 mm wide when opened out, and lemmas with well-developed awns inserted in the lower 1/2 of the lemma.

#### Agrostis breviculmis

Taxon classificationPlantaePoalesPoaceae

Hitchc., U.S.D.A. Bur. Pl. Industr. Bull. 68: 36, pl. 18. 1905

EF62DEF9-C2CF-58FE-8C1E-22E69F351A07

Fig. 3

Trichodium nanum J. Presl, Reliq. Haenk. 1 (4–5): 243. 1830.
Agrostis nana (J. Presl) Kunth, Enum. Pl. [Kunth] 1 (1): 226. 1833.

##### Type.

Peru. Hab. in Peruvia, T. Haenke s.n. [#192 in W0014114 isotype] (holotype: PR; isotypes: BR (BR0000006864774 [image!]), MO (MO2104691 [image!]; MO2104692 [illustration]; MO2114552 [image!]), LE-TRIN (LE-TRIN1627.01 fragm. & fig.), W (W0014113 [image!]; W0014114 [image!])).

##### Description.

**Perennial herbs**, densely tufted, not stooling and without notable lateral tending or ascending rhizomes. **Tillers** intravaginal, without cataphylls. **Culms** 3−12(−15) cm tall, erect to arched, firm, usually with 0 nodes exerted at flowering. **Leaves** basal, glabrous, smooth or scaberulous; **ligules** c. 1 mm long, membranous or slightly scarious, triangular and obtuse, moderately to strongly decurrent with the sheath, abaxial surface smooth or scaberulous; **blades** 1−4(−6) cm long, 0.25−1 mm wide in diameter as folded or rolled, usually convolute, less often strongly conduplicate, recurved, rigid, abaxial surface usually smooth throughout, rarely scaberulous towards the apex, adaxial surface and margins generally scaberulous, sometimes moderately scabrous, apices blunt to slightly broadly naviculate-acute. **Panicles** 1−2.6(−3) cm long, c. 0.05−0.2(−0.6) cm wide, densely congested, spikelike, generally uninterrupted, subincluded in the basal foliage to slightly or moderately exerted, lateral branches with spikelets almost to the base, upper lateral branches short and held close to the central inflorescence axis, central axis and panicle branches scabrous or smooth; **pedicels** 0.9−2 mm long, usually shorter than their spikelets, not obviously dilated at their apex, smooth or scaberulous. **Spikelets** 1.5−2.1 mm long (−2.5 mm long in Bolivia?; Renvoize 1998); **glumes** equal or subequal, lower glume sometimes longer than upper by up to 0.2 mm and slightly to notably wider, 1-veined, lower glume keel scabrous at least in the distal half, prickle hairs coarse and shiny, upper glume keel like that of lower glume or with fewer scabers to sometimes smooth throughout, apices acute; **floret** 2/3−3/4 the length of the glumes; **calluses** pilose, usually with 2 lateral tufts of short hairs; **lemmas** 1.2−1.4 mm long, glabrous, smooth, 5-veined, apex truncate, erose and 4-mucronate, muticous or with a short straight awn to 0.9 mm long, inserted above the middle, not or only briefly surpassing the glume apex, weak and falling easily; **paleas** absent or to 0.3 mm long, < ¼ the length of the lemma; **rachilla** absent; **anthers** 0.5−0.9 mm long.

##### Distribution and ecology.

Amply distributed in the high Andes, from Colombia and Venezuela to Chile and Argentina. Predominantly found in open, grazed páramo and puna vegetation, 3200−4500 m alt.

##### Other specimens examined.

**Colombia**. **Boyacá**: Munic. Chiscas, Vereda Rechiniga, Páramo de la Mesa, páramo perturbado, dominado por *Espeletia* y gramíneas exóticas, con presencia de ganadera caprina, 6.59291N, 72.44541W, 3694 m alt., 3 Mar. 2018, S.P. Sylvester 3096 (FMB, US); Munic. Duitama, Páramo de la Rusia, vía que conduce a vereda de Avendanos, 5.9324667N, 73.0798W, 3726 m alt., 4 Oct. 2017, S.P. Sylvester 3024 (FMB, K, UPTC, US); Munic. Duitama, Páramo La Rusia, Guanenta Alto Río Fonce National Park, top of the ridge Peña Negra just below military base, ridge along the top of a steep rocky landscape, open páramo with *Espeletiacachaluensis* [Rodríguez-Cabeza], edges of the road, 5.58389N, 73.053263W, 3970 m alt., 21 Nov. 2017, M. Vorontsova 2217 (FMB, K, US); Munic. Mongua, Páramo de Ocetá, Valle de Laguna Negra, pajonal frailejonal, terreno inclinado en medio de valle, 5.7066389N, 72.8036111W, 3699 m alt., 29 Nov. 2017; L.E. Cuta-Alarcón 364 (FMB, K, US).

**Figure 3.**
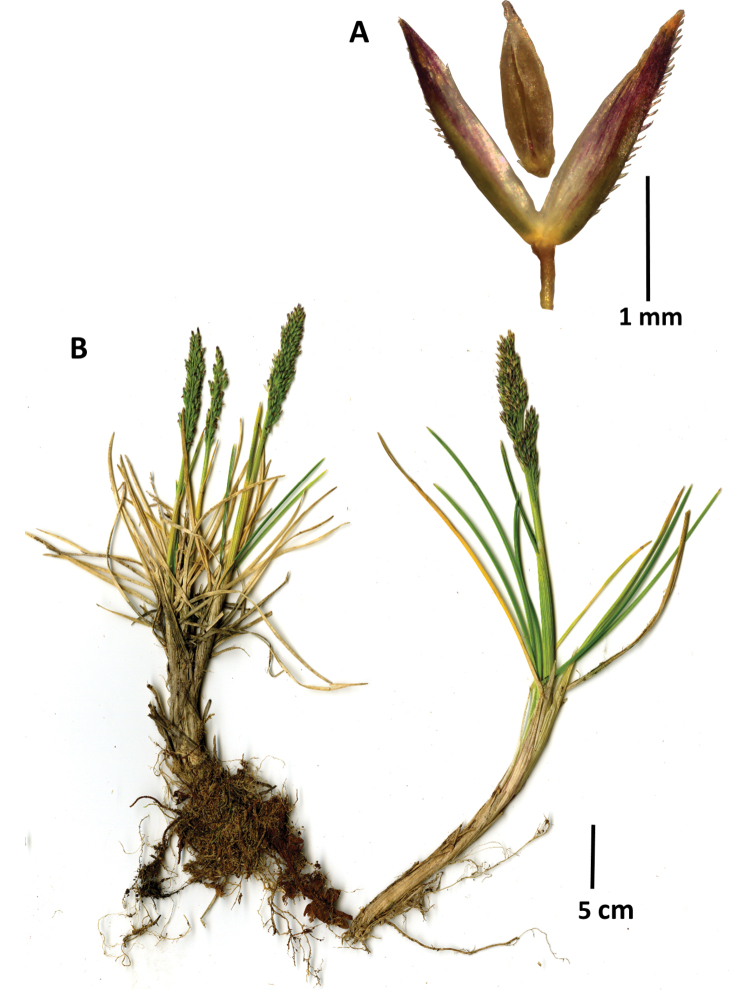
*Agrostisbreviculmis***A** spikelet, glumes in lateral view, floret in ventral view, detached and raised above the glumes **B** whole plant. Images of Cuta-Alarcon 364 (FMB).

##### Similar species.

*Agrostisboyacensis* and *A.meyenii* also have congested spikelike panicles and florets with a small palea and which lack awns. See notes under *A.boyacensis* for how to differentiate these taxa. *Agrostismeyenii* can be principally differentiated by its larger spikelets, 2.2−4.1 mm long, and softer, lax, filiform to flat or folded leaf blades. *Agrostislaegaardii* A.M. Molina & Rúgolo, a recently described species found in Ecuador and the Páramo del Ruiz of the Cordillera Central of Colombia (Palacio et al. in press), also bears very close resemblance. These similarities include: a) plants 3–12 cm tall; b) tillers intravaginal, without notable lateral tending or ascending rhizomes; c) leaf blades convolute, recurved, rigid, 0.5–2 mm wide when opened out, apices blunt or slightly naviculate-acute; d) ligules c. 1 mm long; and e) glumes with coarse shiny prickle hairs along the keel. *Agrostisbreviculmis* can be differentiated from *A.laegaardii* by a) often larger spikelets, (1.7–)2–3.3 mm long (vs. 1.5–2.1(–2.5) mm long in *A.breviculmis*); b) pedicels slightly widened towards the apex and cupuliform (vs. pedicel apex not dilated, truncate, in *A.breviculmis*); c) glumes membranous, standard V-shaped in cross section (vs. chartaceous, narrow V-shaped in cross section in *A.breviculmis*); d) lower glume narrowly ellipsoid (vs. navicular in *A.breviculmis*); e) upper glume scabrous in upper 2/3–3/4 (vs. scabrous in upper ½ in *A.breviculmis*); f) distance between upper glume and floret (0.5–)0.7–1 mm (vs. 0.3–0.5 mm in *A.breviculmis*); g) lemma (1.6–)1.7–2 mm long, lateral veins terminating in 4 aristulas (vs. 1.2–1.4 mm long, lateral veins terminating in 4 mucrons in *A.breviculmis*); h) lemmas with well-developed geniculate awns (1.6–)2.3–3 mm long, inserted in the middle or upper third of the lemma, exerted from the glumes, persistant (vs. awnless or with a short straight awn to 0.9 mm long, inserted above the middle, not or only briefly surpassing the lemma apex, weak and falling easily, in *A.breviculmis*); and i) palea 0.4–0.5 mm long (vs. 0.2–0.3 mm long in *A.breviculmis*).

#### Agrostis capillaris

Taxon classificationPlantaePoalesPoaceae

L., Sp. Pl. 1: 62. 1753

C8AA0E2A-B915-535E-8BFC-2721AE80126F

Fig. 4

Agrostis polymorpha var. capillaris (L.) Huds., Fl. Angl. 1: 31. 1778.
Trichodium capillaris (L.) Roth, Nov. Pl. Sp. 41. 1821.
= Agrostistenuis Sibth., Fl. Oxon. 36. 1794. Agrostiscapillaris Huds., Fl. Angl.: 27. 1762, hom. illeg., non L., 1753. Type: England (not located). Many other heterotypic synonyms.

##### Type.

[Habitat in Europae pratis], Herb. A. van Royen s.n. (lectotype, designated by Widén 1971: 65: L (L0052645 left-hand specimen [image!]); isolectotype: L).

##### Description.

**Perennial herbs**, loosely to densely tufted with short to extensive rhizomes and sometimes with well-developed stolons. **Tillers** extravaginal, with cataphylls present. **Culms** 10−80 cm tall, erect or decumbent at their base, delicate, usually with 2−5 nodes exerted at flowering, smooth or scaberulous. **Leaves** basal and cauline, glabrous, smooth or scaberulous; **ligules** 0.2−1.5(−2.9) mm long, of basal leaves and tillers ≤ 1 mm long, distinctly shorter than wide, of culm leaves 0.5–1.5(–2.9) mm long, membranous, rounded to truncate, not or only slightly decurrent with the sheath, abaxial surface scaberulous to sometimes smooth; **blades** 1−17 cm long, (0.6−)1−5 mm wide as opened out, basal blades and those of tillers often involute and 0.3–0.8 mm wide as rolled, sometimes flat, culm blades generally flat or becoming convolute upon drying, less often involute, usually soft and lax, rarely firm, abaxial and adaxial surfaces and margins smooth or scaberulous. **Panicles** 3−20 cm long, 2.5−8 cm wide, usually open, sometimes partially closed after flowering, lax, usually ovoid to pyramidal, subincluded in the basal foliage to greatly exerted, lateral branches without spikelets in the lower ½, long, ascending or spreading but not held close to the central inflorescence axis, central axis and panicle branches scabrous or smooth; **pedicels** 1−2 mm long, usually shorter than their spikelets, dilated or not at their apex, smooth or scaberulous. **Spikelets** (not including awn, if present) (1.8−)2−2.5(−2.7) mm long; **glumes** subequal, the lower slightly longer than the upper, 1-veined, lower glume keel usually scabrous in the distal half, sometimes scabrous throughout, upper glume keel often smooth throughout, infrequently scabrous in the distal half, apices acute; **floret** 2/3−3/4 the length of the glumes; **calluses** glabrous or with 2 sparse lateral tufts of short hairs; **lemmas** 1.4−2.2 mm long, 3- or 5-veined, glabrous or sometimes pubescent at the base when 5-veined, smooth, apex obtuse or truncate, erose, muticous or with an awn 0.5−3 mm long, inserted above the middle of the dorsal keel, exerted from the glumes or not, straight, flexuose or geniculate, twisted or not, weak and easily falling, usually found only on the 5-veined lemmas; **paleas** 0.6−1.3 mm long, (2/5−)½−¾ the length of the lemma; **rachilla** absent; **anthers** 0.8−1.4 mm long.

**Figure 4.**
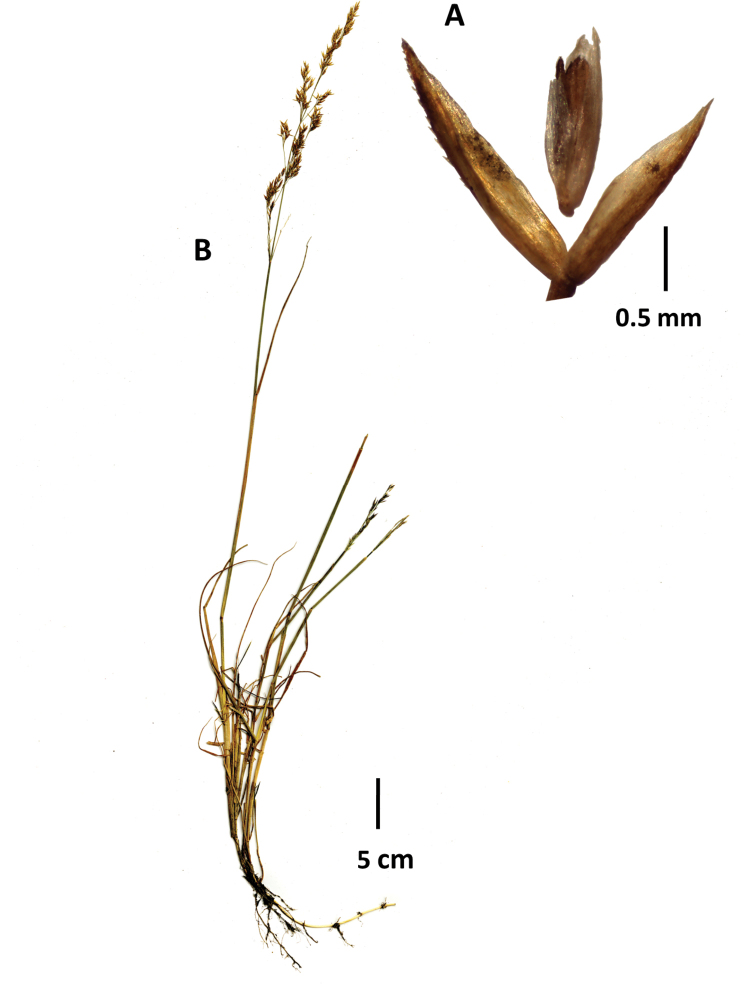
*Agrostiscapillaris***A** spikelet, lateral view, with floret detached and raised above the glumes **B** Whole plant. Images of Sylvester 3021 (FMB).

##### Distribution and ecology.

Eurasian, introduced to the north of South America. Predominantly found in grazed páramo and puna vegetation. *Agrostiscapillaris* is here recorded as a new regional record for the Depto. Boyacá of Colombia. Giraldo-Cañas et al. (2016) mention it as an introduced and cultivated herb based on the record of *A.tenuis* by Hafliger and Scholz (1981). It is likely to be an under-recorded element in many Colombian regions with páramo habitat.

##### Other specimen examined.

**Colombia**. **Boyacá**: Munic. Duitama, Páramo de la Rusia, vía que conduce a vereda de Avendaños, 5.9324667N, 73.0798W, 3726 m alt., 4 Oct. 2017, S.P. Sylvester 3021 (COL, FMB, K, SI, UPTC, US).

##### Notes.

A highly variable species comprising a set of ecologically and genetically distinct populations that are difficult to pull apart taxonomically. The species can hybridize with *A.gigantea* (A.×bjoerkmannii Widen), *A.stolonifera* (A.×murbeckii Fouill. ex P.Fourn.), *A.vinealis* Schreb. (A.×sanionis Asch. & Graebn.), etc., with hybrids exhibiting intermediate characteristics and all being sterile (Cope and Gray 2009). *Agrostiscapillaris* is often planted in the Neotropics as part of a lawn mix with other species, or for livestock feed.

##### Similar species.

*Agrostiscastellana* Boiss. & Reut., which has not been recorded for Colombia but is found introduced and naturalized further south in Argentina and Chile (Rúgolo de Agrasar 2012). The only certain characters for distinguishing *A.castellana* from *A.capillaris* are that, in *A.castellana*, the terminal spikelets of the inflorescence branches have a distinctly pubescent upper dorsal surface of the lemma, at least towards the margins, while they are usually glabrous or pubescent only at the base in *A.capillaris*. The panicles are also contracted, lanceolate to narrowly oblong after flowering in *A.castellana*, while being open with spreading branches after flowering in *A.capillaris*. Other characters, such as awn presence, are too variable to be useful in separating these species. Furthermore, these species can also hybridize to produce A.×foilladei P. Fourn., which has been reported from seed mixtures for amenity grassland in the UK (Cope and Gray 2009). *Agrostisgigantea*, another closely related species which can usually be readily distinguished by its greater size and relatively longer ligules, does have some smaller variants with thinner leaves that superficially resemble *A.capillaris*, but the ligule will always settle the identity.

#### Agrostis foliata

Taxon classificationPlantaePoalesPoaceae

Hook. f., Bot. Antarct. Voy. I. (Fl. Antarct.). 1: 95. 1845

E5120C2B-4C20-53C7-8E39-585AD3D56D80

Fig. 5

= Agrostisnigritella Pilg., Bot. Jahrb. Syst. 25 (5): 713–714. 1898. Type: Ecuador. Chimborazo: in páramos montis Antisana ad Cerro de la Media Luna, 4400 m alt., no date, M.A. Stuebel 231 (holotype: [not found]; isotype: US (US75324 fragm.)).
= Agrostisstuebelii Pilg., Bot. Jahrb. Syst. 25 (5): 714. 1898. Type: Colombia. Tolima: in monte ignivomo Tolima fere ad nivis limitem adscendens, no date, Stuebel 198 (lectotype, designated here: US ex B (US00902474!); isolectotype: US ex B (US00902473!)). Syntype: Colombia. In monte ignivomo Purace copiose, ubi usque adcineris conum reperiteur, no date, Stuebel 298 (SI ex US (SI000509 [image!], US ex B (US75325)).

##### Type.

Colombia [Ecuador]. Crescit in cacumine monte Pichincha [On Pichincha at the limits of perpetual snow], 15676 ft [4778 m alt. based on protologue; 15000 ft written on isotypes], no date [21 Jan. 1856 on GH isotype], W. Jameson 229 (holotype: K; isotypes: GH (GH00221375 [image!]), P (P00740550 [image!]), US (US843246 fragm. ex K)).

##### Description.

**Perennial herbs**, tussock-forming or laxly to densely tufted, usually with short ascending rhizomes. **Tillers** extravaginal and intravaginal, with cataphylls usually present. **Culms** 15−30 cm tall, erect, rigid and thickened, with 0 nodes exerted at flowering, smooth or scaberulous. **Leaves** usually more-or-less evenly spread along the culm, sometimes congested basally, glabrous, usually densely scabrous; **ligules** 3−8 mm long, of upper culm 4−8 mm long, usually scarious (at least in part), triangular and obtuse to acute, moderately to strongly decurrent with the sheath, abaxial surface scaberulous to scabrous; **blades** 3−12 cm long, 2−6 mm wide when opened out, flat or folded, sometimes somewhat involute towards their apices, straight or slightly recurved, subcoriaceous to coriaceous, surfaces and margins usually densely scabrous throughout, apices naviculate-acute. **Panicles** 5−12.7 cm long × 1−1.7(−2.5) cm wide, densely congested, spikelike, sometimes interrupted towards the base, usually exerted from the basal foliage or sometimes subincluded, lateral branches with spikelets usually present almost to the base, upper lateral branches short and held close to the central inflorescence axis, central axis and panicle branches moderately to densely scabrous; **pedicels** 0.7−4 mm long, usually shorter than their spikelets, not obviously dilated at their apex, scabrous. **Spikelets** (not including awn) 3–4.2 mm long; **glumes** equal or subequal, 1-veined, keels smooth or lightly scabrous, surfaces smooth, apices acuminate; **floret** usually c. ½ the length of the glumes, rarely slightly longer; **calluses** pilose with 2 sparse tufts of short hairs on the lateral sides; **lemmas** 1.5−2 mm long, glabrous, smooth, 5-veined, apex truncate, denticulate, awned, awn 2−2.8 mm long, inserted in the lower ½ of the dorsal keel, exerted from the glumes, geniculate, twisted, persistant; **paleas** absent or 0.1−0.2 mm long, < ¼ the length of the lemma; **rachilla** absent; **anthers** (0.5−)0.6−0.8(−1) mm long.

**Figure 5.**
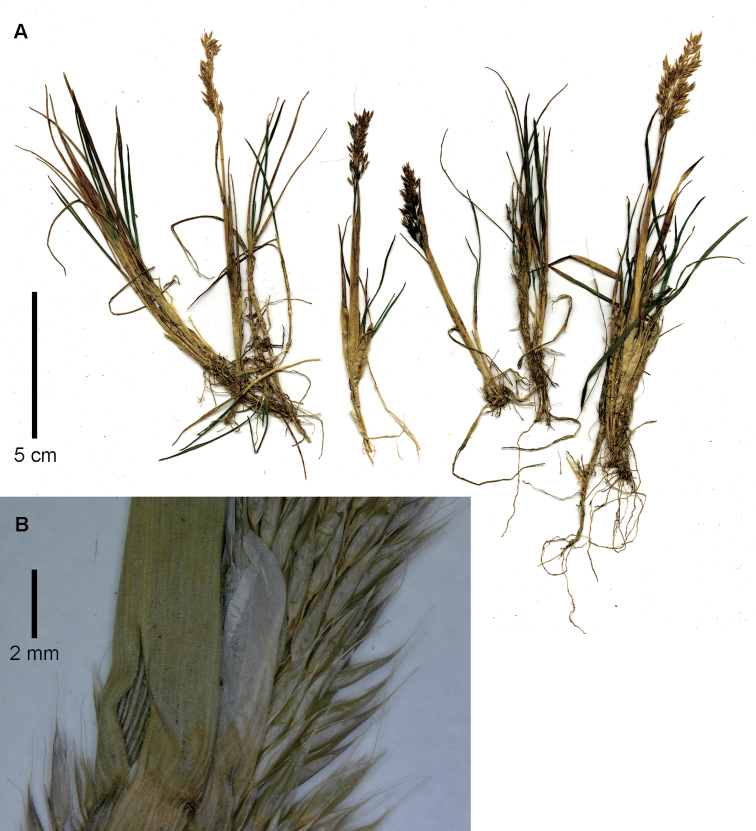
*Agrostisfoliata***A** whole plant **B** flag ligule and portion of inflorescence. Image **A** Sylvester 3151 (FMB) **B** Grubb 306 (US2304908).

##### Distribution and ecology.

Colombia, Venezuela, Ecuador, Peru. High-elevation superpáramo and subnival vegetation in the puna grasslands of Peru. In Boyacá, found in superpáramos, > 4300 m alt.

##### Other specimens examined.

Colombia. **Boyacá**: Munic. Chiscas, Páramo de Chacaritas, arribando a la morrena, 6.61779N, 72.38899W, 4354 m alt., 4 Mar. 2018, S.P. Sylvester 3130 (FMB, K, US); Munic. Chiscas, Páramo el Penon, Chiscas, 6.63012N, 72.40073W, 4172 m alt., vegetación de pajonal frailejonal, páramo húmedo, 3 Mar. 2018, S.P. Sylvester 3151 (FMB, K, US); Sierra Nevada del Cocuy, 4600 m alt., tufted grass in loose soil, 6 Aug. 1957, P.J. Grubb 306 (US2304908; US2433280); Sierra Nevada del Cocuy, alto valle de Las Lagunillas, [6.3469N, 72.33W], 4000–4300 m alt., 12 Sept. 1938, J. Cuatrecasas 1466 (US2780332); 1467 (US1772999); 1474 (US1773004; US2855551); Sierra Nevada del Cocuy, Páramo Cóncavo, Superpáramo, abrigo rocoso, denominado Cueva de los Hombres, 3 km aprox. al N del morro Pulpito del Diablo, [6.3995N, 72.3122W], 4350 m alt., gramínea cerca del abrigo, asociada con *Senecioguicanensis*, 28 Feb. 1973, A.M. Cleef 8609 (US2785677); Sierra Nevada del Cocuy, Quebrada Bocatoma, superpáramo 2 km al E de la Laguna Pintada, [6.4795N, 72.3167W], 4280 m alt., playa arenosa húmeda con gramíneas, *Ditrichum* sp. y algas terrestres, gramínea muy común, 5 Oct. 1972, A.M. Cleef 5862 (US3207543); Sierra Nevada del Cocuy, Quebrada Bocatoma, superpáramo, [6.4795N, 72.3167W], 4310 m alt., playa arenosa húmeda con gramíneas y cianófitos terrestres de la lagunita central, gramínea común, 04 Oct. 1972, A.M. Cleef 5792 (US2797551); **Caldas**: Nevado del Ruiz, superpáramo, vallecitos poco húmedos, Arenales 1 km al SW del Refugio cerca del empalme de la carretera, [4.8956N, 75.3508W], 4680 m alt., 18 Mar. 1972, A.M. Cleef 2398 (US2785739).

##### Notes.

Two syntypes of *A.stuebelii* were noted in Pilger’s protologue of *Agrostisstuebelii*. We lectotypify the name on the best material at US that includes one flowering and one non-flowering plant, with the bases of the plants well preserved. We were unable to locate the two duplicates of the lectotype at B, from which the US material was taken.

##### Similar species.

See notes under *A.boyacensis*. *Agrostistolucensis* can sometimes have leaf blades wider than usual (up to 5 mm wide when opened out), but can usually be differentiated by a) the flag ligules being shorter, 2–4(–6.2) mm long (vs. 4–8 mm long in *A.foliata*); b) the spikelets often being shorter, 2−3(−3.6) mm long (vs. 3–4.2 mm long in *A.foliata*); c) florets usually over half the length of the glumes (vs. usually c. ½ the length of the glumes, rarely slightly longer in *A.foliata*); and d) primary panicle branches often shorter, 0.5–1.5 cm long, (vs. up to 7 cm long in *A.foliata*), among other characteristics.

#### Agrostis cf. imberbis

Taxon classificationPlantaePoalesPoaceae

Phil. Anales Univ. Chile 94: 11. 1896

4E7CFCB1-729C-5040-8D8C-94AC584BC6C6

Fig. 6

= Agrostisstenophylla Phil., Anales Univ. Chile 94: 10. 1896. Type: Chile. Bío-Bío: Baños de Chillán, Jan. 1878, Philippi s.n. [“146” in Herb. R. A. Phillippi s.d.; “No. 18 (II)” on SGO000000043 isotype] (holotype: SGO-PHIL (SGO-PHIL146); isotypes: BAA (BAA00000030 [image!]), BM (BM000938541 [image!]), SGO (SGO000000043 [image!], SGO000000061 [image!], SGO000000062 [image!]), US (US00156498! fragm. ex SGO-PHIL146), W (W19160040646 [image!])).
= Agrostisscotantha Phil., Anales Univ. Chile 94: 16. 1896. Type: Chile. Araucanía: La Cueva, Jan. 1887, C. Rahmer s.n. [“183” in Herb. R. A. Phillippi s.d.] (holotype: SGO-PHIL (SGO-PHIL183); isotypes: BAA (BAA00000728 [image!] fragm. ex SGO-PHIL183, BAA00000729 [image!] fragm. ex B), SGO (SGO000000055 [image!], SGO000000056 [image!]), US (US00156493! fragm. ex SGO-PHIL183), W (W19160040640 [image!])).
= AgrostismoyanoiSpeg.var.plicatifolia Speg., Anales Mus. Nac. Buenos Aires 7: 189. 1902. Type: Argentina. Chubut: Corcovado, Oct. 1901, N. Illin 2550 (holotype: LP (LP001364 [image!]; isotypes: BAA (BAA00000023 [image!])).

##### Type.

Chile. Ñuble: Valle de las Nieblas, Chillán, Jan. 1877, sin col. s.n. [“185” in Herb. R. A. Phillippi s.d.] (holotype: SGO-PHIL (SGO-PHIL185); isotypes: BAA (BAA00001345 [image!]), BM (BM000938537 [image!]), SGO (SGO000000042 [image!], SGO000000044 [image!], US (US00156439! fragm. ex SGO-PHIL-185), W (W19160040633 [image!])).

##### Description.

**Perennial herbs**, laxly to densely tufted, sometimes stooling, with laterally tending or ascending pseudostolons, or rhizomatous with rhizomes ascending or vertical. **Tillers** intravaginal and extravaginal, with cataphylls usually present. **Culms** (7−)24−60(−65) cm tall, usually decumbent at their base or slightly creeping and rooting from the nodes, rarely completely erect, firm, with (0−)1−2 nodes exerted at flowering, smooth or scaberulous. **Leaves** usually mostly basal in first seasons growth, becoming mostly cauline as plant ages, glabrous, usually scaberulous with undeveloped scabers throughout, sometimes notably scabrous throughout; **ligules** 2.5−6.7(−10) mm long, of upper culm (2.8−)3−6.7(−10) mm long, usually scarious (at least in part), acute to acuminate, sometimes fimbriate, strongly decurrent with the sheath, abaxial surface scabrous; **blades** 2.8−9(−11) cm long, 0.5−1 mm wide in diameter as rolled or folded, convolute, involute, or conduplicate, rigid, abaxial surface smooth to scaberulous, adaxial surface lightly scabrous on the veins, apices usually naviculate-acute. **Panicles** (3−)6−12.5(−20) cm long, (0.5−)2−6 cm wide, open to slightly contracted when young, usually ovoid, slightly to greatly exerted from the basal foliage, lateral branches without spikelets near their base and for a long distance, long and ascending or spreading to becoming divaricate at maturity but not held close to the central inflorescence axis, central axis and panicle branches scaberulous; **pedicels** (0.9−)1.5−3.5 mm long, often slightly longer than their spikelets or sometimes shorter, dilated or not at their apex, scaberulous. **Spikelets** (2.3−)2.5−2.7(−3.5) mm long; **glumes** equal or subequal, similar or lower glume slightly wider and longer than the upper, 1-veined, keels usually scabrous in the distal ½−¾, upper glume sometimes completely smooth, apices acute to acuminate; **floret** usually 3/4 the length of the glumes or slightly longer; **calluses** glabrescent, with very sparse short hairs; **lemmas** 1.6−1.8(−2.5) mm long, glabrous, lightly scaberulous throughout or just in distal 1/2, sometimes with just undeveloped scabers as pustulate bases, 5-veined, apex obtuse or truncate, denticulate, muticous, mucronate, or sometimes with a short straight awn to 0.5 mm long, inserted in the middle or upper 1/3 of the lemma and not surpassing the glumes, weak and falling easily; **paleas** usually c. 0.2 mm long, < ¼ the length of the lemma; **rachilla** absent; **anthers** 1−1.2(−2) mm long.

**Figure 6.**
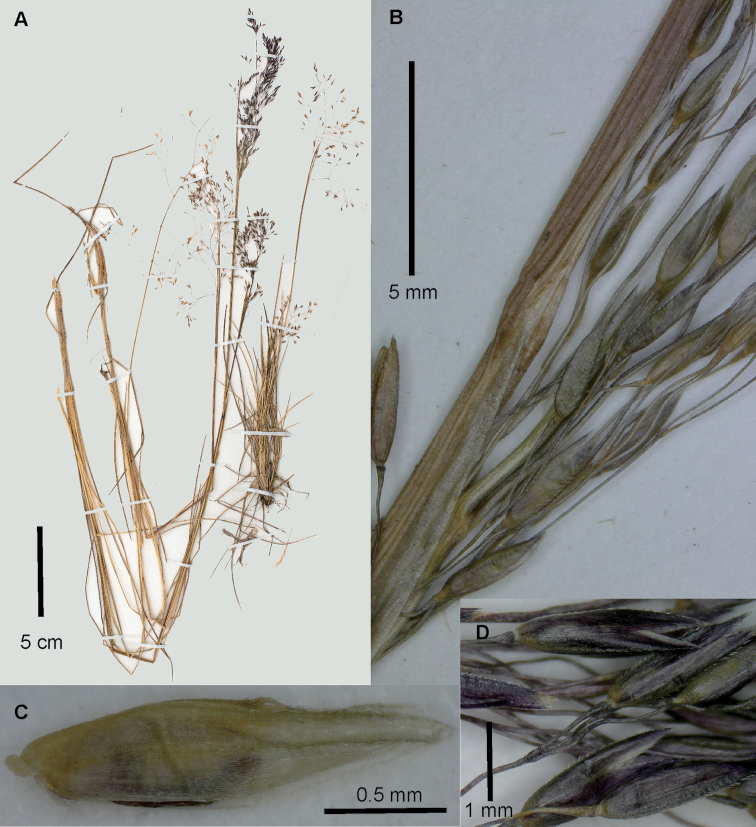
Agrostiscf.imberbis**A** whole plant **B** flag ligule and portion of inflorescence **C** floret, lateral view **D** portion of inflorescence with close-up of spikelets. Images of Cleef 6821 (US2785719), courtesy of United States National Herbarium (US).

##### Distribution and ecology.

Agrostiscf.imberbis is a new record for Colombia and páramo vegetation in general, and is not found in the most recent checklist for Colombia (Giraldo-Cañas et al. 2016), the páramo checklist (Luteyn 1999) or checklists for Ecuador (Jørgensen and Ulloa-Ulloa 1994; Jørgensen and León-Yánez 1999), Venezuela (Hokche et al. 2008; Bono 2010), or Costa Rica (Morales-Quirós 2003) which host páramo vegetation. The species was previously considered to be restricted to Argentina and Chile (Rúgolo de Agrasar 2012), but has also been recorded for Bolivia (Jørgensen et al. 2014) and Peru (Davidse et al. 1993; Soreng et al. 2003 and onwards; W3TROPICOS-Peru Checklist 2020; Óscar Tovar unpubl. data).

##### Additional specimens examined.

**Colombia**. **Boyacá**: Munic. Belén, Páramo de La Rusia, near Páramo El Consuelo, unprotected private land, somewhat disturbed páramo grazed by horses and rodents, dominated by *Espeletiaboyacensis* [Cuatrec.], 6.02146N, 72.5718W, 3832 m alt., 22 Nov. 2017, M. Vorontsova 2228 (US); Munic. Chiscas, Páramo el Penon, Chiscas, borde de bosque de *Polylepis*, 6.59582N, 72.44284W, 3771 m alt., 5 Mar. 2018, S.P. Sylvester 3159 (K, SI, US); Munic. Chiscas, Páramo de Chacaritas, pajonal frailejonal, cercano al cerro de Chacaritas, con fuerte grado de inclinacion, 6.63108N, 72.39815W, 4082 m alt., 5 Mar. 2018, S.P. Sylvester 3144 (FMB, K, SI, UPTC, US); Munic. Duitama, Páramo de La Rusia, NW-N de Duitama, [5.9281N, 73.0936W], 3600 m alt., aislada, vertiente seca con *Calamagrostiseffusa* [*Paramochloaeffusa*] predominante, asociada con *Espeletiaboyacensis* y *Acaenacylindristachya*, gramínea muy abundante, 7 Dec. 1972, A.M. Cleef 6821 (US2785719); Munic. Duitama, Páramo de La Rusia, vía que conduce a vereda de Avendanos, páramo semi-perturbado, pastado por cabras y quemado regularmente hasta hace 1 año, 5.95011N, 73.09097W, 3795 m alt., 1 Oct. 2017, S.P. Sylvester 3014 (COL, FMB, K, SI, US); 5.9324667N, 73.0798W, 3726 m alt., 4 Oct. 2017, S.P. Sylvester 3029 (FMB, US); S.P. Sylvester 3031 (K, US); [Munic. El Cocuy and Soatá,] between Soatá and Cocuy, Páramo del Alto del Escobal, [6.3514N, 72.5483W], 3750 m alt., 8 Oct 1938, J. Cuatrecasas 1232 (US1772922).

##### Notes.

Specimens studied from Boyacá match most characteristics of *A.imberbis* apart from sometimes the leaf blade abaxial surfaces being scaberulous, albeit with silica ‘pustules’ present throughout that sometimes developed into short hooks, and spikelets sometimes being shorter, to 2.3 mm long. Rúgolo de Agrasar (2012) mentions leaf blades can be smooth in *A.imberbis* under exceptional circumstances. Characters that are diagnostic for the species, such as narrow, rolled (convolute or involute) or conduplicate, rigid blades, long acuminate scarious ligules, usually large spikelets > 2.5 mm long, and glabrescent calluses, were all present on the specimens studied from Boyacá. Given its disjunct distribution and slight differences in morphology, coupled with knowledge that other widespread species such as *A.perennans* are actually numerous evolutionarily distinct taxa (Konstantin Romaschenko pers. communication), we refer to this species with ‘cf.’ to highlight that it needs to be checked in a molecular framework.

The species has a variable level of panicle congestion depending on the stage of maturity.

##### Similar species.

*Agrostissubrepens*, a species described from Mexico and whose presence in Colombia is uncertain (see notes on *A.subrepens* under ‘Excluded species’ at the end of the taxonomic treatment), and A.cf.imberbis share similar morphologies. This includes the habit being decumbent and stooling, appearing rhizomatous or pseudostoloniferous, blades being convolute and rigid, and panicles usually large and open with spikelets that have a similar shape and size with lemmas lacking notable awns, ligules being strongly decurrent with the sheath and usually scarious (at least in part), and anthers > 1 mm long. While culm and panicle size are generally larger and anthers are often shorter in *A.subrepens*, some overlap occurs with *A.imberbis*, with ligule shape and size seeming to be the only solid character to differentiate them. *Agrostissubrepens* has shorter (< 2.2 mm long), obtuse upper culm ligules while A.cf.imberbis has acute to acuminate upper culm ligules (2.8−)3−6.7(−10) mm long.

*Agrostisperennans* s.l. can sometimes have involute and rigid basal blades, but the upper culm blades are flat and lax, unlike those of A.cf.imberbis, which are rigid and convolute, involute, or strongly conduplicate throughout. The ligules in *A.perennans* s.l. are also usually shorter, with ligules of basal leaves and tillers 0.5−2.5 mm long, while those of A.cf.imberbis are > 2.5 mm long. Ligule apices of *A.perennans* s.l. are also truncate or obtuse-triangular, unlike the acute or long acuminate ligules of A.cf.imberbis.

*Agrostisvinealis* Schreb., a species not recorded for Colombia, but found introduced and naturalized in Argentina and Chile (Soreng and Peterson 2003; Rúgolo de Agrasar 2012), bears similarities in terms of overall habit, being notably rhizomatous and having mainly basal leaves and an exerted open panicle that becomes congested after flowering, as well as other similarities such as the palea being reduced and awns sometimes absent (but usually with a persistent, geniculate and twisted, awn to 4 mm long, inserted near the base of the lemma). *Agrostisvinealis* usually has leaf blades that are flat towards the base, although sometimes these are involute and setaceous, and scabrous throughout or at least on the adaxial surface. It can also be differentiated by the ligules being often shorter, of the tillers 1−2.5 mm long, of the upper culms 1−4(−5) mm long, with apices rounded or bluntly pointed (vs. of the tillers > 2.5 mm long, of the upper culms (2.8−)3−6.7(−10) mm long, with apices acute to acuminate in A.cf.imberbis).

#### Agrostis mertensii

Taxon classificationPlantaePoalesPoaceae

Trin., Linnaea 10 (3): 302. 1836

DED1619C-E72D-5713-9424-FFF7019A50FD

Fig. 7

Agrostis laxiflora var. mertensii (Trin.) Griseb., Fl. Ross. 4 (13): 442. 1852.
Agrostis canina var. mertensii (Trin.) Kuntze, Revis. Gen. Pl. 3[3]: 338. 1898.
= Agrostisboliviana Mez, Repert. Spec. Nov. Regni Veg. 18 (1–3): 1. 1922. Type: Bolivia. Pinos bei Tarija, 3000 m alt., 22 Jan. 1904, K.A.G. Fiebrig 2821 (lectotype, designated by Rúgolo de Agrasar (2012: 115): BAA (BAA00000014 [image!]); isolectotypes: BAA (BAA00000211 [image!] fragm. ex K), G (G00099216 [image!]), K (K000308377 [image!]), L (L0819974 [image!]; L0819973 [image!]); syntypes: Bolivia. Calderillo, 3000 m alt., Mar. 1904, K.A.G. Fiebrig 2905, BAA (BAA00000013 [image!]; BAA00000210 [image!]), E (E00373832 [image!]), G (G00099217 [image!]), GH (GH00221373 [image!]; GH00221374 [image!]), K (K000308376 [image!]), S (S05-10054 [image!])).
= Agrostisgelida Trin., Mém. Acad. Imp. Sci. Saint-Pétersbourg, Sér. 6, Sci. Math., Seconde Pt. Sci. Nat. 6 (2, Bot.): 343. 1841. Type: Peru. Andibus de Pasco, [ad nives aeternas], E.F. Poeppig s.n. (holotype: LE-TRIN (LE-TRIN1613.01); isotype: US (US75321 fragm.)). Many other heterotypic synonyms.

##### Type.

USA. Alaska, 1829, D. Mertens s.n. (lectotype, designated by Widen (1971: 52): LE-TRIN (LE-TRIN1622.01, plant 1); isolectotypes: BAA (BAA00001355 [image!] fragm. ex LE-TRIN), S (S-G-263 [image!] fragm. ex LE-TRIN)).

##### Description.

**Perennial herbs**, tufted, sometimes with short lateral tending rhizomes present or stooling with pseudostolons present. **Tillers** extravaginal, with cataphylls present. **Culms** 20−65 cm tall, erect, ascendant, or geniculate at the base, delicate and slender, with 0−2(−3) nodes exerted at flowering, smooth. **Leaves** mostly cauline but with basal leaves present, glabrous, smooth throughout or finely scaberulous on the blade adaxial surface and margins; **ligules** (0.6−)2−6 mm long, membranous or scarious, obtuse to acuminate, not or slightly to moderately decurrent with the sheath, abaxial surface smooth or scaberulous; **blades** 10−13 cm long, 1−3 mm wide when opened out, usually flat, soft and lax, less often involute and semi-rigid, tiller blades tending to be more involute and thin, semi-rigid, smooth throughout or finely scaberulous on the adaxial surface and/or margins, apices acute to acuminate. **Panicles** 5−15 cm long, c. 4−16 cm wide, open when flowering and mature, congested when immature, lax, ovoid to pyramidal, slightly to usually greatly exerted from the basal foliage, lateral branches without spikelets in the lower ½, long, ascending, spreading, to somewhat divergent and not held close to the central inflorescence axis at flowering or maturity, adpressed when young, central axis and panicle branches scabrous or smooth; **pedicels** 1−6(−15) mm long, sometimes shorter to usually much longer than their spikelets, not or slightly dilated at their apex, smooth or scaberulous. **Spikelets** (not including awn) (2.2−)2.5−3(−4) mm long; **glumes** unequal, the lower slightly longer than the upper by up to c. 0.5 mm, 1-veined, keels smooth to scabrous throughout or more commonly scabrous in the upper half, apices acute; **floret** usually 2/3−3/4 the length of the glumes; **calluses** lightly pilose with 2 sparse lateral tufts of short hairs; **lemmas** 1.7−2 mm long, glabrous, smooth, 5-veined, apex obtuse to acute, 2-dentate, 2−4 mucronate, awned, awn (1.8−)2.5−4.5 mm long, inserted dorsally in the middle or lower third, exerted from the glumes, flexuose to geniculate, twisted, persistant; **paleas** absent or 0.2−0.5 mm long, < ¼ the length of the lemma; **rachilla** absent; **anthers** 0.5−1 mm long.

**Figure 7.**
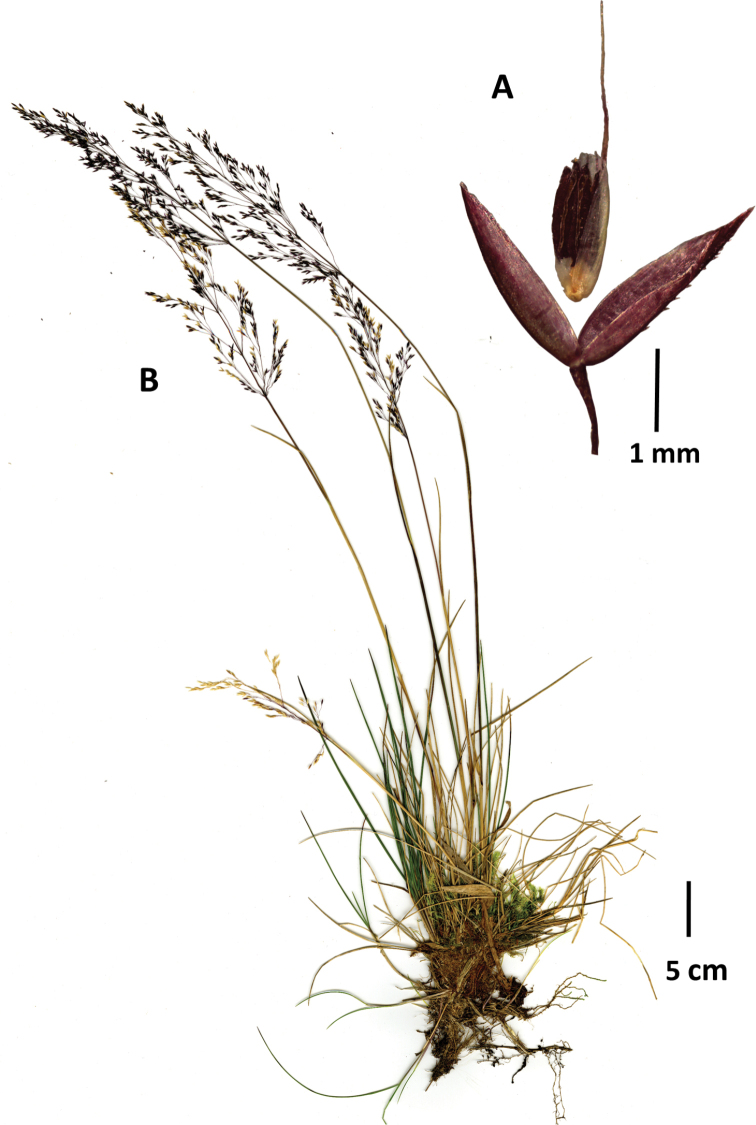
*Agrostismertensii***A** spikelet, lateral view, with floret detached and raised above the glumes **B** whole plant. Images of Cuta-Alarcon 365 (FMB).

##### Distribution and ecology.

Exhibits a disjunct distribution, being found in very cold Arctic and sub-Arctic areas of the Northern Hemisphere (i.e. North America, Europe and Asia), and also in the high Andes of Venezuela, Colombia, Peru, Bolivia, Argentina and Chile (Soreng et al. 2003 and onwards; Rúgolo de Agrasar 2012). It has most likely been introduced and naturalized in South America.

##### Other specimens examined.

**Colombia**. **Boyacá**: Munic. Arcabuco, Páramo El Valle, Vereda el Carmen, páramo muy húmedo dominado por *Chusquea*, pastoreo natural, 5.75425N, 73.3830278W, 3430 m alt., 15 Nov. 2017, S.P. Sylvester 3067b (COL, FMB, K, US); Munic. Duitama, Páramo de Agueros, vía que conduce a vereda de Avendanos, 5.91464N, 73.07114W, 3445 m alt., 28 Oct. 2017, S.P. Sylvester 3047 (FMB, K, US); Munic. Duitama, Páramo de Agueros, semi disturbed páramo in Agueros reserve above ridge- along path running through reserve, 5.91653N, 73.07164W, 3445 m alt., 29 Oct. 2017, S.P. Sylvester 3052 (FMB, K, UPTC, US); Munic. Duitama, Páramo de La Rusia, Guanenta Alto Río Fonce National Park, top of the ridge Peña Negra just below military base, ridge along the top of a steep rocky landscape, open páramo with *Espeletiacachaluensis*, no signs of grazing, 5.58389N, 73.053263W, 3970 m alt., 21 Nov. 2017, M. Vorontsova 2198 (FMB, K, US); Munic. Duitama, Páramo de La Rusia, Fina Betania, vereda El Carmen on other side of river, 5.95333N, 73.10864W, 3445 m alt., 30 Oct. 2017, S.P. Sylvester 3060 (FMB, K, US); Munic. Duitama, Páramo de La Rusia, vía que conduce a Vereda de Avendanos, páramo semi-perturbado, pastado por cabras y quemado regularmente hasta hace 1 año, 5.95011N, 73.09097W, 3795 m alt., 1 Oct. 2017, S.P. Sylvester 3004 (COL, FMB, K, US); Munic. Duitama, valley between Guantiva and La Rusia, between Susacon and Onsaga, 2 km towards Onzaga, 6.10091N, 72.48263W, 3292 m alt., 24 Nov. 2017, M. Vorontsova 2304a (FMB, K, US); Munic. Mongua, Páramo de Ocetá, Valle de Laguna Negra, pajonal frailejonal, terreno inclinado en medio del valle, 5.70664N, 72.80361W, 3699 m alt., 29 Nov. 2017, L.E. Cuta-Alarcón 365 (FMB, K, UPTC, US); Munic. Mongui, Páramo de Ocetá, Sector La Pedrisca, Vereda Vallado, potrero con 20 años de abandono dominado por *Senecio* y *Espeletiaboyacensis*, y gramíneas exóticas, 5.69969N, 72.80892W, 3751 m alt., 30 Nov. 2017, L.E. Cuta-Alarcón 377 (FMB, K, SI, US); Munic. Sotaquira, protected area of Páramo El Valle, day 2 East ridge walk, somewhat disturbed patch of open páramo between patches of *Chusqueatessellata* [Munro], dominated by *Espeletiaboyacensis*, limited natural grazing, 5.44436N, 73.22089W, 3717 m alt., 16 Nov. 2017, M. Vorontsova 2155 (FMB, K, US).

##### Notes.

This species is highly variable in terms of its habit, the form of its leaf blades (flat or involute) and ligule (short and obtuse to long and acuminate), and the form of the panicle (panicle branches adpressed and congested when young while open when mature). The combination of open panicle (when mature), lemma with a dorsally inserted awn, and a minute or absent palea are diagnostic for this species.

##### Similar species.

*Agrostisperennans* s.l., which can be principally differentiated by the absence of an awn, or if awn present, this being inserted in the upper half of the dorsal surface of the lemma or subapically and to 0.5 mm long. *Agrostispittieri* Hack., considered endemic to Costa Rica by Pohl (1980) and Morales-Quirós (2003), also bears close similarity to *A.mertensii* and may be placed as a synonym of the latter in future research. Hokche et al. (2008) and Dorr (2014) record *A.pittieri* for Venezuela, with Dorr (2014) stating *A.pittieri* can be tentatively differentiated by having linear and narrow panicles with green spikelets and lemmas 1.9−2 mm long, while *A.mertensii* has broadly ovate to lanceolate panicles with purple spikelets and lemmas 2−2.5 mm long. However, these characters’ overlap in specimens studied from throughout the range of *A.mertensii*.

#### Agrostis perennans

Taxon classificationPlantaePoalesPoaceae

(Walter) Tuck. s.l., Amer. J. Sci. Arts 45: 44. 1843

638D5321-DB6B-5F9E-BE33-2D3FCBF41B79

Fig. 8

Cornucopiae perennans Walter, Fl. Carol. [Walter] 74. 1788.
Agrostis cornucopiae Sm., Gentleman’s Mag. 59 (2): 873. 1789, nom. illeg. superfl., also Smith in Frasier, Short Hist. 2,pl. Nov. 25. 1789.
Agrostis cornucopiae Lam., Tabl. Encycl. 161. 1791, nom. illeg. superfl.
Agrostis elegans (Walter) Salisb., Prodr. Stirp. Chap. Allerton 25. 1796, nom. illeg. superfl.
Agrostis anomala Willd., Sp. Pl. 1: 370. 1797, nom. illeg. superfl.
Trichodium decumbens (Walter) Michx., Fl. Bor.-Amer. (Michaux) 1: 42. 1803, nom. illeg. superfl.
Trichodium perennans (Walter) Elliott, Sketch Bot. S. Carolina [Elliott] 1: 99. 1816.
Agrostis scabra var. perennans (Walter) Alph. Wood, Class-book Bot. (ed. 1861) 774. 1861.
= Agrostisfasciculata (Kunth) Roem. & Schult., Syst. Veg., ed. 15 bis [Roemer & Schultes] 2: 362. 1817. Vilfafasciculata Kunth, Nov. Gen. Sp. [H.B.K.] 1: 139. 1816. Type: Ecuador. Pichincha, May, F.W.H.A. von Humboldt & A.J.A. Bonpland s.n. (holotype: P (P00669402 [image!]; isotypes: HAL (HAL0106915 [image!]), LE-TRIN (LE-TRIN1610.01 fragm. ex P), P (P00740549 [image!]; P00740548 [image!]), US (US556249 fragm. ex P)).
= Agrostishumboldtiana Steud., Nomencl. Bot. [Steudel], ed. 2. i. 40. 1840. Agrostispulchella Kunth, Enum. Pl. 1: 223. 1833, hom. illeg., non Roth, 1817, nom. superfl. Agrostiselegans (Kunth) Roem. & Schult., Syst. Veg., ed. 15 bis [Roemer & Schultes] 2: 362. 1817, hom. illeg., non. Salisb. 1796. Vilfaelegans Kunth, Nov. Gen. Sp. [H.B.K.] 1: 139. 1816. Type: Ecuador. [Crescit in planitie Cochapamba, in regione temperata regni Quitensis, alt. 1340 hexap. Floret Majo] Herbier de Amerique equatoriale, A.J.A. Bonpland & F.W.H.A. von Humboldt 3010 (holotype: P (P00669401 [image!]); isotypes: BAA (BAA-Col. typus 4256), BM (BM000938529 [image!]; BM000938530 [image!]), LE-TRIN (LE-TRIN1644.01 ex hrbr. Humb.), P (P00740586 [image!]; P00740588 [image!]; P00740587 [image!])).
= Agrostisweberbaueri Mez, Repert. Spec. Nov. Regni Veg. 18 (1–3): 1. 1922. Type: PERU. Huacapistana and Monson, Weberbauer s.n. (holotype: [not found]; isotypes: [not found]). Many other heterotypic synonyms.

##### Type.

USA. South Carolina: Richmond County, Fort Jackson Military Reservation, found at the intersection of Cut Off Rd. and Fire Break 49, 11 July 1995, K.B. Kelly, Jr. & J.B. Nelson 254 (neotype, designated by Ward (2007: 1099): GH (GH00247994 [image!])).

##### Description.

**Perennial herbs**, laxly to densely tufted, sometimes stooling or with short ascending pseudostolons that have the appearance of rhizomes on herbarium sheets. **Tillers** extravaginal, with cataphylls present. **Culms** (21−)33−64(−100) cm tall, erect or decumbent to subgeniculate at their base, delicate to fairly firm, with 0−2(−3) nodes exerted at flowering, smooth to rarely scaberulous. **Leaves** mainly basal early in the flowering season but tending to become mostly cauline with maturity, glabrous, smooth or scaberulous; **ligules** 0.5−5(−7) mm long, of basal leaves and tillers 0.5−2.5 mm long, of upper culm 3−5(−7) mm long, truncate to obtuse-triangular, slightly to strongly decurrent with the sheath, abaxial surface scabrous to scaberulous, rarely smooth; **blades** (3−)6−15 cm long, (1–)1.5−3.5(−5) mm wide, usually flat or conduplicate and lax to slightly firm, sometimes involute and rigid in the basal leaves, smooth throughout or scaberulous on margins and sometimes surfaces, apices acute. **Panicles** (3.5−)10−22 cm long, 2−11 cm wide, open, lax, ovoid to pyramidal, slightly to usually greatly exerted from the basal foliage, lateral branches without spikelets near their base and for a large distance, long, ascending, spreading, to somewhat divergent and not held close to the central inflorescence axis, central axis and panicle branches smooth or lightly scaberulous; **pedicels** 1−4.5 mm long, usually longer than their spikelets, not or slightly dilated at their apex, smooth or lightly scaberulous. **Spikelets** (not including awn, if present) (1.5−)1.8−2.5(−3.2) mm long; **glumes** subequal, 1-veined, keels scabrous in the distal ½−1/3, apices acute; **floret** usually 1/2−2/3 the length of the glumes, rarely longer; **calluses** lightly pilose with 2 sparse lateral tufts of short hairs; **lemmas** 1.5−2 mm long, glabrous, smooth, 5-veined, apex acute to more-or-less truncate, denticulate, muticous, mucronate or exceptionally with a short awn to 0.5 mm long (to 1.4−1.9 mm long in Argentina; Rúgolo de Agrasar 2012), inserted in the upper (½) 1/3 of the dorsal keel, usually not or rarely only slightly surpassing the glumes, straight, not twisted, weak and falling easily; **paleas** absent or to 0.5 mm long, usually < ¼ the length of the lemma; **rachilla** absent; **anthers** 0.7−1 mm long.

**Figure 8.**
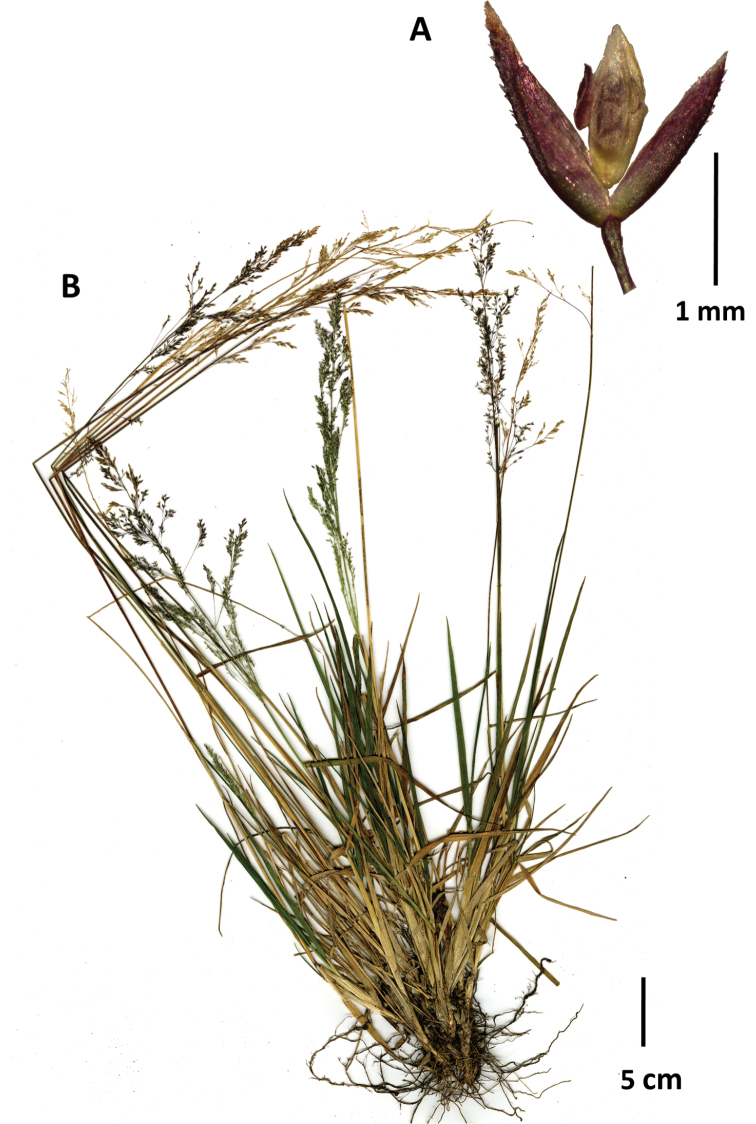
*Agrostisperennans* s.l. **A** spikelet, glumes in lateral view, with floret in dorsal view, detached and raised slightly above the glumes **B** whole plant. Images of Vorontsova 2247 (FMB).

##### Distribution and ecology.

Stretching from Alaska, Canada and USA, through Central America and the Caribbean, to Argentina and Chile of South America. The species has an exceptionally large ecological amplitude, being found in maritime dunes, wetlands, and grasslands from sea level to > 4000 m alt.

##### Other specimens examined.

**Colombia**. **Boyacá**: Munic. Belén, Páramo de La Rusia, near Páramo El Consuelo, unprotected private land, somewhat disturbed páramo grazed by horses and rodents, with *Espeletiaboyacensis* and *E.discoides*, near path, 6.02415N, 72.57289W, 3831, 22 Nov. 2017, M. Vorontsova 2247 (FMB, K, SI, UPTC, US); Munic. Duitama, Páramo de Agueros. junto a la casa, muy perturbado, pastizales junto a la plantación de pinos, 8.91069N, 73.07219W, 3377 m alt., 28 Oct. 2017, S.P. Sylvester 3039 (FMB, K, SI, UPTC, US); Munic. Duitama, Páramo de La Rusia, Guanenta Alto Río Fonce National Park, top of the ridge Peña Negra just below military base, ridge along the top of a steep rocky landscape, 5.58389N, 73.053263W, 3970 m alt., open Páramo with *Espeletiacachaluensis*, no signs of grazing, 21 Nov. 2017, M. Vorontsova 2203 (US); Munic. Duitama, Páramo de la Rusia, Vereda El Carmen, 5.95333N, 73.11019W, 3445 m alt., 30 Oct. 2017, S.P. Sylvester 3064 (FMB, K, SI, US); Munic. Duitama, Páramo de la Rusia, vía que conduce a vereda de Avendaños, 5.95011N, 73.09097W, 3795 m alt., 1 Oct. 2017, S.P. Sylvester 3002 (COL, FMB, K, SI, US); 5.93246N, 73.0798W, 3726 m alt., 4 Oct. 2017, S.P. Sylvester 3036 (COL, K, SI, UPTC, US); Munic. Sotaquira, protected area of Páramo El Valle, páramo just above riverine forest, 5.4605N, 73.21507W, 3220 m alt., 17 Nov. 2017, M. Vorontsova 2161 (K, SI, US); Sierra Nevada del Cocuy, Alto Valle Lagunillas, páramo pantanoso al sur de la Laguna Cuadrada, [6.3619N, 72.3345W], 4060 m alt., 26 Sep. 1972, A.M. Cleef 5579 (US2785750). **Nariño**: Páramo del Tábano, alto de la Cordillera, entre Pasto y El Escano, vertiente occidental, [1.1756N, 77.1867W], 3200 m alt., 11 Jan. 1941, J. Cuatrecasas 11908-A (US1798782).

##### Notes.

*Agrostisperennans* appears to be a ‘grab-bag’ of many different taxa that come out in many different places amongst other taxa of *Agrostis* in molecular phylogenies (Konstantin Romaschenko pers. communication), with the species complex needing urgent revision. Specimens found in Boyacán páramos correspond in almost all characteristics with *Agrostisperennans* s.s. apart from sometimes being found with shorter spikelets (c. 1.5−2 mm long) and, what appear to be, short ascending rhizomes/pseudostolons, with these characteristics also found on type material of *A.fasciculata*, which is here considered a synonym of *A.perennans* until comprehensive systematic research is undertaken on the species complex. Cataphyllous extravaginal shoots are found on the neotype of *A.perennans* as well as other type material of species considered synonyms of *A.perennans*, e.g. *A.decumbens* E. Fourn., with it plausible that these ‘rhizomes’ are short pseudostolons that arise from growing through moss or between rocks.

Type specimens and specimens from North America, Central America, and northwest South America have lemmas without awns or with awns under-developed, to 0.5 mm long, straight and easily falling. Specimens noted by Rúgolo de Agrasar (2012: 119) to have awns to 1.4 mm long, or exceptionally to 1.9 mm long, may be a distinct taxon, but further research is needed to ascertain this.

##### Similar species.

*Agrostismertensii*, which is principally differentiated in having a well-developed, flexuose to geniculate, twisted awn (1.8−)2.5−4.5 mm long, inserted in the middle or lower third of the lemma, and which is greatly exerted from the glumes (vs. unawned or exceptionally with a short straight awn to 0.5 mm long, inserted in the upper (½) third of the dorsal keel, usually not or rarely only slightly surpassing the glumes in *A.perennans* s.l. specimens from northwest South America, Central America and North America).

*Agrostissubrepens*, here considered to be excluded from Departamento Boyacá and possibly Colombia (see notes under ‘Excluded species’ *A.subrepens*), sometimes bears certain similar, but not equal, characteristics such as a) plants often stooling with notable pseudostolons and appearing long rhizomatous on herbarium sheets; b) culms 50−100 cm tall, slightly creeping or decumbent at their base but erect towards their apex; c) panicles open, lax, 5–10 cm wide; d) lemma unawned or awn to 0.5 mm long and straight, not twisted, inserted in the middle or upper 1/3 of the lemma or subapical; e) palea absent or < ¼ the length of the lemma. *Agrostissubrepens* can be differentiated from *A.perennans* s.l. by a) upper culm ligules 1−2.2 mm long, obtuse (vs. 3−5(−7) mm long, truncate to obtuse-triangular in *A.perennans* s.l.) b) leaf blades 0.5–1 mm wide in diameter as rolled or folded, convolute, involute, or conduplicate, rigid, surfaces scabrous (vs. leaf blades (1–)1.5−3.5(−5) mm wide, usually flat or conduplicate and lax to slightly firm, sometimes involute and rigid in the basal leaves, smooth throughout or scaberulous on margins and sometimes surfaces in *A.perennans* s.l.); anthers 1−1.3 mm long (vs. 0.7−1 mm long in *A.perennans* s.l.)

*Agrostisturrialbae*, here considered to be excluded from Departamento Boyacá and possibly Colombia, bears similarities and may possibly be part of the *A.perennans* s.l. species complex (see notes under ‘Excluded species’ *A.turrialbae*).

*Agrostisvinealis*, of Eurasian origin which is not recorded for Colombia, but found introduced and naturalized in Argentina and Chile (Soreng and Peterson 2003; Rúgolo de Agrasar 2012), bears certain similarities (see also comments regarding this species under A.cf.imberbis). It can be differentiated by having a) lemmas usually with a persistent, geniculate and twisted awn to 4 mm long, inserted near the base (sometimes absent); b) rhizomes conspicuous, long, lateral tending, and usually covered in bracts; c) panicles contracted and rather dense before and after flowering.

#### Agrostis stolonifera

Taxon classificationPlantaePoalesPoaceae

L., Sp. Pl. 1: 62. 1753

7142DF34-C240-55CD-B980-925206686C0D

Fig. 9

##### Type.

[Habitat in Europa], Herb. A. van Royen s.n. (lectotype, designated by Widén 1971: 77: L (L0059234 [image!])).

Many heterotypic synonyms.

##### Description.

**Perennial herbs**, generally creeping, usually extensively stoloniferous with long stolons to 200 cm long, less often with short stolons, rhizomes usually absent. **Tillers** extravaginal, with cataphylls present. **Culms** 15−100 cm tall, erect or decumbent at their base, delicate to fairly firm, nodes usually held within sheaths with 0(−3) exerted at flowering, usually smooth to rarely scaberulous. **Leaves** mainly cauline, glabrous, scaberulous; **ligules** 1−6.5(−8) mm long, of basal leaves and tillers 1−3 mm long, of upper culm 2−6.5(−8) mm long, rounded to truncate, not or slightly to moderately decurrent with the sheath, abaxial surface scaberulous; **blades** 2−26 cm long, 1−8 mm wide, flat or sometimes folded, soft and lax, surfaces and margins scaberulous to scabrous, apices acute. **Panicles** 2−20(−32) cm long, 1−16 cm wide, open and lax or contracted after flowering, ovoid to pyramidal, slightly to usually greatly exerted from the basal foliage, lateral branches naked in the lower 1/3−1/4 or with spikelets present to the base, long, ascending, spreading, to somewhat divergent and not held close to the central inflorescence axis at or before flowering, held close to the central rachis at maturity, central axis and panicle branches scabrous or very rarely smooth; **pedicels** 0.5−2.5 mm long, usually shorter than their spikelets, dilated or not at their apex, scabrous. **Spikelets** (not including awn, if present) 1.8−3 mm long; **glumes** subequal or unequal, the lower slightly longer than the upper by up to c. 0.5 mm, 1-veined, lower glume keel usually scabrous in the distal half, upper glume often smooth throughout, apices acute; **floret** usually 2/3−3/4 the length of the glumes; **calluses** with 2 sparse lateral tufts of short hairs; **lemmas** 1.2−2.2(−2.5) mm long, (3-) 5-veined, glabrous or rarely pubescent at the base with hairs 0.1−0.2 mm long, smooth, apex obtuse or truncate, erose, muticous or rarely with an awn 0.5−3 mm long, usually inserted above the middle of the dorsal keel, exerted or not from the glumes, straight, flexuose or geniculate, twisted or not, weak and falling easily; **paleas** 0.7−1.3(−1.6) mm long, (2/5−)½−¾ the length of the lemma; **rachilla** absent; **anthers** 0.9−1.6 mm long.

**Figure 9.**
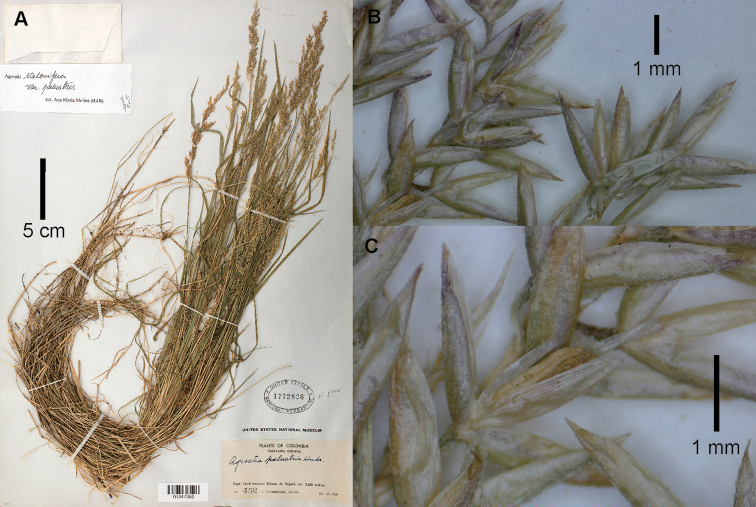
*Agrostisstolonifera***A** whole plant **B** inflorescence, close-up **C** spikelet, close-up, lateral view. Image **A** Cuatrecasas 452 (US1772826) **B** García-Barriga 6 (US2115003).

##### Distribution and ecology.

Widespread and cosmopolitan or Eurasian origin, introduced to South America from Europe. While no specimens of *A.stolonifera* were verified from Departamento Boyacá at US or encountered during extensive fieldwork in the region, the species is cited for Boyacá in the checklist (Giraldo-Cañas et al. 2016) and specimens were found at US from the neighboring Departamento Cundinamarca in the Cordillera Oriental meaning it likely that the species occurs in Boyacá.

##### Other specimens examined.

**Colombia**. **Cundinamarca**: Sabana de Bogota, 2600 m alt., 29 Dec. 1938, J. Cuatrecasas 452 (US1772826); Tunjuelo, granja experimental “La Picota”, [4.5331N, 74.1117W], 2600 m alt., 3 Feb. 1933, H. García-Barriga 6 (US2115003).

#### Agrostis tolucensis

Taxon classificationPlantaePoalesPoaceae

Kunth, Nov. Gen. Sp. [H.B.K.] 1: 135. 1816

9FED202A-6589-55E4-8A38-CF45E18212D9

Fig. 10

Agrostis tolucensis Willd. ex Steud., Syn. Pl. Glumac. 1: 164. 1855[1854], nom. inval.
= Agrostisglomerata (J. Presl) Kunth, Enum. Pl. [Kunth] 1: 219. 1833. Vilfaglomerata J. Presl, Reliq. Haenk. 1 (4–5): 239. 1830. Type: Peru. [Huánuco] [Hab. in montanis Peruviae huanoccensibus], [1791; 1891 written on W isotype], T.P.X. Haenke s.n. [#196 written on W isotype] (holotype: [not found]; isotypes: HAL (HAL0106916 [image!]), PRC (PRC450953 [image!]), US (US00589472 fragm.), W (W0025326 [image!])).
= Agrostishoffmannii Mez, Repert. Spec. Nov. Regni Veg. 18: 3. 1922. Type: Costa Rica. Irazu (holotype: [not found]; isotypes: [not found]).
= Agrostisnanavar.andicola Pilg., Bot. Jahrb. Syst. 37 (5): 505. 1906. Type: Ecuador. Chimborazo: Chimborazo, N-Seite, Páramo-Region, 4500 m alt., July 1903, H. Meyer 146 (syntypes: JE (JE00020226 [image!]; JE00020228 [image!])); Napo: Antisana, in frigidis alpinis, 4600 m alt., July 1903, H. Meyer 145 (syntypes: JE (JE00020225 [image!]; JE00020227 [image!])).
= Agrostisnanavar.aristata Griseb., Abh. Königl. Ges. Wiss. Göttingen 24: 294. 1879. Type: Argentina. Salta: Umgebungen des Nevado del Castillo [around the Nevado del Castillo], 10−15000 ft. [3048−4572 m alt.], 19–23 Mar. 1873, P.G. Lorentz & G.H.E.W. Hieronymus 82 (holotype: GOET (GOET006541 [image!]); isotypes: B, BAA (BAA00000724 [image!] fragm. ex B; BAA00000723 [image!] fragm. ex B), CORD (CORD00004693 [image!]), W (W19160036588 [image!]; W19160036664 [image!])).
= Agrostisvirescens Kunth, Nov. Gen. Sp. [H.B.K.] 1: 135. 1816. Type: Mexico. Toluca, Sep., F.W.H.A. Humboldt & A.J.A. Bonpland s.n (holotype: P (P00669395 [image!]); isotypes: LE-TRIN (LE-TRIN1668.01; LE-TRIN1668.02), P (P00136912 [image!])).

##### Type.

Mexico. [prope Toluca et Islahuaca], F.W.H.A. Humboldt & A.J.A. Bonpland s.n. (holotype: P (P00669394 [image!]); isotypes: B (B-W1704), BAA (BAA00000235 [image!] fragm. ex P), BM, K (microfiche), LE-TRIN (LE-TRIN1660.01), P (P00136914 [image!]; P00136915 [image!]; P00740426 [image!]), US (US00156505! fragm. ex P)).

##### Description.

**Perennial herbs**, tussock-forming or laxly to densely tufted, usually with short ascending rhizomes. **Tillers** extravaginal and intravaginal, with cataphylls usually present. **Culms** (3−)5.5−51(−80) cm tall, erect or arching, fairly firm, with 0(−2) nodes exerted at flowering, smooth or rarely scaberulous. **Leaves** mostly basal or more-or-less evenly spread along the culm, glabrous, smooth or scabrous; **ligules** 2−4(−6.2) mm long, of upper culm usually longer than those of lower culm and tillers, truncate to triangular, moderately to strongly decurrent with the sheath, abaxial surface usually scabrous, rarely scaberulous or smooth; **blades** 2.5−19 cm long, 1−3(−5) mm wide, at least in the upper culm filiform, folded or flat, usually lax to sometimes firm, sometimes basal and tiller blades involute or convolute and firm to rigid, smooth throughout or scabrous only on the margin and sometimes veins, apices blunt or slightly naviculate-acute. **Panicles** (1−)2−15 cm long, 0.1−1.5 cm wide, moderately to densely congested, sub-spikelike or spikelike, sometimes interrupted towards the base, subincluded in the basal foliage or slightly to greatly exerted, lateral branches with spikelets almost to the base, upper lateral branches short and held close to the central inflorescence axis, central axis and panicle branches scabrous; **pedicels** 0.7−3(−4.5) mm long, usually shorter than their spikelets, not or slightly dilated at their apex, scabrous. **Spikelets** (not including awn, if present) 2−3(−3.6) mm long; **glumes** equal or subequal, 1-veined, lower glume keel and surface usually scabrous at least in the distal half, infrequently smooth throughout, upper glume keel and surface scabrous for almost their entirety to at least in the distal half or surface sometimes smooth throughout, apices acute or acuminate; **floret** ½−2/3(−3/4) the length of the glumes; **calluses** lightly pilose with 2 sparse tufts of short hairs on the lateral sides; **lemmas** 1.4−2.8 mm long, glabrous, smooth, 5-veined, apex truncate, denticulate, usually awned, awn 2−3.5 mm long, inserted in the lower, middle, or upper 1/3 of the dorsal keel, exerted from the glumes, geniculate, twisted, very rarely muticous or with a short straight awn, < 1 mm long, inserted in the upper half of the lemma (see notes below regarding *A.glomerata*); **paleas** absent or 0.1−0.2 mm long, < ¼ the length of the lemma; **rachilla** absent; **anthers** 0.5−1 mm long.

**Figure 10.**
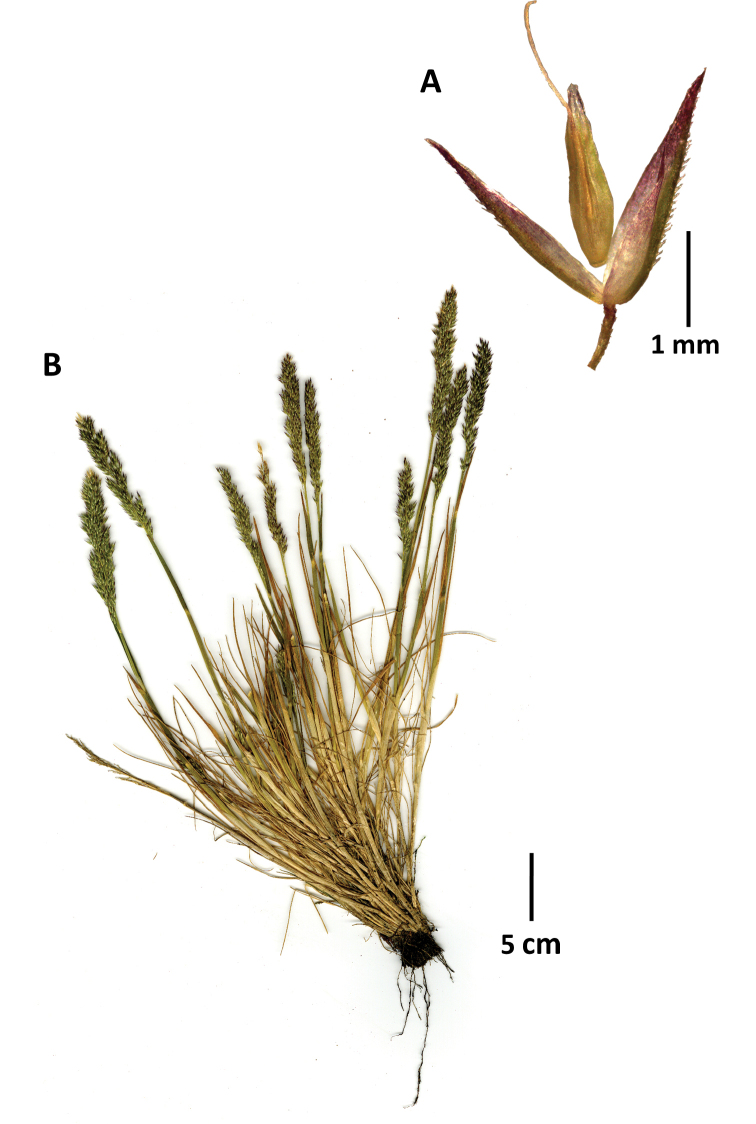
*Agrostistolucensis***A** spikelet, glumes in lateral view, floret in dorsal view, detached and raised above the glumes **B** whole plant. Images of Cuta-Alarcon 376 (FMB).

##### Distribution and ecology.

Mexico, Guatemala, Costa Rica, Panama, Colombia, Venezuela, Ecuador, Peru, Bolivia, Chile, Argentina. High-elevation open grasslands and forests, 2700−4900 m alt.

##### Other specimens examined.

**Colombia**. Sin loc., 1760–1808, J.C. Mutis 5521 (US1561906). **Boyacá**: Munic. Arcabuco, Páramo El Valle, 5.7445833N, 73.37118W, 3744 m alt., 16 Nov. 2017, S.P. Sylvester 3081 (FMB, K, US); Munic. Arcabuco, Páramo El Valle, just below high point of ridge to the W of site 14, 5.75436N, 73.38722W, 3747 m alt., páramo seco dominado por *Espeletiabarclayana* [Cuatrec.], 16 Nov. 2017, S.P. Sylvester 3083 (K, US); Munic. Belén, Páramo de La Rusia, border with Santander, Boqueron El Consuelo, unprotected private land, 6.03598N, 72.57056W, 3975 m alt., páramo with limited grazing by horses and rodents with *Espeletiabrachyaxiantha* [S. Díaz] and *E.annemariana* [Cuatrec.], steep slope on rock outcrop, 23 Nov. 2017, M. Vorontsova 2260 (FMB, K, SI, US); Munic. Belén, Páramo de La Rusia, near Páramo Le Consuelo, unprotected private land, somewhat disturbed páramo on top of the ridge, 6.02416N, 72.57242W, 3894 m alt., grazed by horses and rodents, with *Espeletiaboyacensis*, 22 Nov. 2017, M. Vorontsova 2231 (K, UPTC, US); Munic. Chiscas, Páramo de Chacaritas, asociado a rocas de 4 m de altura, 6.62227N, 72.3904W, 4192 m alt., 4 Mar. 2018, S.P. Sylvester 3115 (US); Munic. Chiscas, Páramo de Chacaritas, límites entre páramo y superpáramo, 6.62865N, 72.3944W, 4064 m alt., 4 Mar. 2018, S.P. Sylvester 3104 (FMB, K, US); Munic. Chiscas, Páramo El Peñon, Chiscas, 6.63012N, 72.40073W, 4172 m alt., 5 Mar. 2018, S.P. Sylvester 3152 (UPTC); Munic. Chiscas, Páramo El Peñon, Chiscas, 6.62876N, 72.40283W, 4255 m alt., vegetación de pajonal frailejonal, páramo húmedo, 5 Mar. 2018, S.P. Sylvester 3153 (FMB, K, US); Munic. Mongua, Páramo de Ocetá, Valle de Laguna Negra, 5.69525N, 72.79133W, 3694 m alt., 29 Nov. 2017, L.E. Cuta-Alarcón 355 (FMB, K, UPTC, US); Munic. Mongui, Vereda Vallado, Sector La Pedrisca, Páramo de Ocetá, 5.69969N, 72.80891W, 3751 m alt., 30 Nov. 2017, L.E. Cuta-Alarcón 376 (FMB, US). **Cauca**: Macizo Colombiano, Valle de las Papas, alrededores de Valencia, [1.8831N, 76.6828W], 2910 m alt., 11 Sep.-1 Oct. 1958, Idrobo, Pinto & Bischler 3682A (US2540528). **Cundinamarca**: Páramo de Chisaca, [4.2747N, 74.2006W], 3750–3962 m alt., 5 Oct. 1966, T.R. Soderstrom 1312 (US3136708).

##### Notes.

Although in Bolivia (Renvoize 1998) and austral South America (Rúgolo de Agrasar 2012) *A.tolucensis* is mentioned to be notably rhizomatous, lateral tending rhizomes were not notable on specimens from the páramos of Boyacá, which had short, ascending rhizomes/pseudostolons and formed small tussocks or were laxly to densely tufted. Type specimens and original material of *A.tolucensis* and other species now considered synonyms of *A.tolucensis*, i.e. *Agrostisglomerata*, A.nanavar.andicola, A.nanavar.aristata, and *A.virescens*, also had either short ascending rhizomes or lacked notable rhizomes, but with all having obvious extravaginal cataphyllous shoots.

Discrepancy was sometimes also found in the form of the leaf blades, with specimens encountered in Boyacá, Colombia, and Venezuela sometimes having more rigid, convolute to folded, often recurved, basal leaf blades instead of the lax and filiform leaf blades more common to this species. These specimens did usually still have flat upper culm blades to help differentiate them from e.g. *A.laegaardii* (see notes on similar species below). While Renvoize (1998) and Rúgolo de Agrasar (2012) state the blades in *A.tolucensis* to be flat, folded, or filiform and generally lax, the type material also has fairly firm blades which are narrow and folded to slightly convolute and sometimes cylindrical in outline. *Agrostisnana*, here considered a synonym of *A.tolucensis*, also has more convolute, curved and rigid leaf blades.

##### Similar species.

See notes under *A.foliata* for how to differentiate from that species. *Agrostisbreviculmis* and *A.laegaardii* bear similarities in having mainly basal, narrow, usually convolute, rigid, leaf blades and densely congested spikelike panicles that are usually short (< 5 cm long), and spikelets with an absent or reduced palea < ¼ the length of the lemma. *Agrostistolucensis* principally differs from these by the leaf blades being usually laxer and folded or flat, at least in the upper culm. However, as noted above, specimens can be found with convolute basal blades that makes distinguishing these species more difficult. In these instances, *A.tolucensis* can be differentiated from *A.breviculmis* by its larger spikelets 2−3(−3.6) mm long (vs. 1.5−2.1(−2.5 in Bolivia?; Renvoize 1998) mm long in *A.breviculmis*), usually the presence of a well-developed geniculate and twisted awn inserted dorsally usually in the lower third of the lemma (lemmas very rarely muticous or with a short straight weak awn, < 1 mm long, inserted in the upper ½; see notes on *A.glomerata* below) (vs. lemmas muticous or with a short straight weak awn inserted above the middle in *A.breviculmis*), panicles generally wider, 1−15 mm wide (vs. 0.5−2(−6) mm wide in *A.breviculmis*), and short prickle hairs on the glume keels (vs. coarse and shiny in *A.breviculmis*). While *A.laegaardii* has characters of often larger spikelets and dorsally inserted awn, *A.tolucensis* can be differentiated from *A.laegaardii* by its extravaginal cataphyllous shoots and distinct ascending rhizomes with rhizome internodes usually > 7 mm long (vs. purely intravaginal innovations, without cataphyllous shoots or distinct ascending rhizomes, rhizome internodes < 2 mm long in *A.laegaardii*), generally wider panicles, 1−15 mm wide (vs. 0.5−2(−6) mm wide in *A.laegaardii*), and short prickle hairs on the glume keels (vs. coarse and shiny in *A.laegaardii*). Specimens can sometimes have laxer inflorescences that could lead to confusion with *A.mertensii*, but this species does not form small tussocks.

*Agrostismeyenii*, a species known from drier high-elevation puna grassland and pampa of Argentina, Chile and Bolivia (Renvoize 1998; Rúgolo de Agrasar 2012), is similar in its overall appearance, usually being tufted and with rhizomes, having similar ligules and filiform or flat leaf blades, and having a condensed spikelike panicle with spikelets of similar size. *Agrostistolucensis* can usually be differentiated from *A.meyenii* by the presence of an awn inserted in the lower third of the lemma, 2−3.5 mm long, twisted and bent and exerted from the glumes (vs. muticous or, if awn present, inserted in the middle or upper third of the lemma, to 1.2 mm long, straight or slightly flexuous in *A.meyenii*). However, specimens akin to *A.glomerata*, a species described from Peru and here considered a synonym of *A.tolucensis*, can sometimes be found in Colombia, albeit not in the Cordillera Oriental, which have muticous lemmas or with a short straight awn inserted in the upper half of the lemma (e.g. Idrobo 3882a, Mutis 5521). These can be differentiated from *A.meyenii* by the plants being generally taller, 20−60 cm tall, condensed panicles often > 10 cm long that are often interrupted and with the central inflorescence axis notably wider compared to the lateral branches, and the pedicels, panicle branches, and sometimes central inflorescence axis, being notably scabrous. The blades of these are variable and can be filiform to flat and to 5 mm wide. The characteristic of notably scabrous pedicels, panicle branches, and sometimes central inflorescence axis of *A.tolucensis* are considered to be key in differentiating these from *A.meyenii*, which are usually smooth or exceptionally lightly scaberulous.

#### Podagrostis

Taxon classificationPlantaePoalesPoaceae

(Griseb.) Scribn. & Merr. Contr. U.S. Natl. Herb. 13 (3): 58. 1910

F1D83F71-4474-594E-8738-DE6D077F814F

Agrostis sect. Podagrostis Griseb. Fl. Ross. 4 (13): 436. 1852.

##### Type.

Agrostiscaninavar.aequivalvis Trin. (lectotype, designated by Hitchcock 1920: 127).

##### Description.

**Perennials. Leaves** mainly basal; **ligules** membranous. **Inflorescence** a panicle, lax and open to contracted and spikelike. **Spikelets** 1-flowered, disarticulating above the glumes, laterally compressed; **glumes** as long as the spikelet, equal or subequal, persisting on the plant after the florets have fallen; lower glume 1- or 3-veined; upper glume 1- (2-) or 3-veined; **floret** subequalling to equaling the apex of the glumes; **lemmas** membranaceous, often slightly thicker than the glumes, dorsally rounded, 3- or 5-veined, lateral veins not evident to distinct; **paleas** well-developed, reaching from (2/3) ¾ to subequaling the lemma, keels obscure to distinct, glabrous, usually smooth; **calluses** rounded, blunt, usually glabrous, or with two short lateral tufts of hairs to 0.5 mm long in some species, abaxially smooth; **rachilla** prolongation present, varying from rudimentary to 2/3 the floret in length, glabrous or sometimes with short strict hairs to 0.3 mm long emerging only from the apex, smooth or scaberulous. **Flowers** perfect; **anthers** 3 in number, 0.4–2.2 mm long. **Caryopses** hard.

#### Podagrostis trichodes

Taxon classificationPlantaePoalesPoaceae

(Kunth) Sylvester & Soreng, PhytoKeys 148: 42. 2020

282FAA03-8E1D-5D69-B3DA-C5222B548A4B

Fig. 11

Vilfa trichodes Kunth, Nov. Gen. Sp. [H.B.K.] 1: 139. 1816.
Agrostis trichodes (Kunth) Roem. & Schult., Syst. Veg. (ed. 15 bis) 2: 361. 1817.
Aira trichodes (Kunth) Spreng., Syst. Veg., ed. 16 [Sprengel] 1: 276. 1824.
= Agrostisbogotensis Hack., Repert. Spec. Nov. Regni Veg. 8: 518. 1910. Type: Colombia. S. Cristobal prope Bogota [près de Bogota], [2500–3000 m alt.], 13 July 1908, *F. Apolliniaire s.n.* (holotype: W (W19160027256 [image!]); isotypes: BM (BM000938528 [image!]), MPU (MPU027104 [image!]), SI (SI000495 [image!] fragm. ex US), US (US75365 fragm.)).

##### Type.

Peru. Crescit in crepidinibus Andium Peruvianum justa Montan, Santa Cruz et Guambos, alt. 1350 hexap. [2469 m alt.], floret Augusto, *F.W.H.A. Humboldt & A.J.A. Bonpland s.n.* (holotype: P; isotypes: HAL (HAL0106929 [image!]), US (US75364! fragm. ex P)).

##### Description.

**Perennial herbs**, forming short dense tufts, with the basal mats reaching c. 4–11 cm tall and inflorescences well-exerted from the basal foliage. **Tillers** intravaginal, without cataphylls. **Culms** 7–20(–30) cm tall, erect, simple, delicate, with 0(−1) nodes exerted at flowering, smooth. **Leaves** mostly basal, glabrous, finely to densely scabrous; **ligules** 0.7–1.7(–2.5) mm long, of basal leaves and tillers 0.7–1.2 mm long, of upper culm leaves generally longer, truncate to obtuse, slightly to usually strongly decurrent with the sheath, abaxial surface smooth or rarely scaberulous; **blades** 1–4 cm long, 0.3–0.4 mm wide in diameter, involute or convolute, acicular to capillaceous and filiform, usually curved, rigid, apices acute. **Panicles** 2–5(–6) cm long, 1–2(–3) cm wide, lax and open, ovoid, slightly to usually greatly exerted from the basal foliage, lateral branches with spikelets in the distal 1/3, the lower 2/3 naked, long, ascending to patent, not held close to the central inflorescence axis, central axis and panicle branches usually scaberulous or sometimes smooth; **pedicels** 1–2 mm long, usually longer than the length of the spikelets, not or slightly dilated at their apex, smooth to usually lightly scabrous. **Spikelets** 1–1.5 mm long; **glumes** equal or subequal, the lower often slightly longer than the upper, 1-veined, keels scabrous just in the distal 1/3 to throughout their length, surfaces smooth a scabrous distally, apices obtuse to acute; **floret** almost equaling the length of the glumes or slightly shorter; **calluses** glabrous; **lemmas** 1–1.5 mm long, glabrous, moderately to densely scabrous (‘smooth’ possibly mentioned by Tovar 1993!), sometimes granulose, apex obtuse, faintly to strongly 5-veined, awn lacking or to 0.5 mm long, straight, inserted medially or in the upper half of the lemma, not surpassing the glumes; **paleas** (0.7–)0.9–1.3 mm long, usually reaching from ¾ to subequaling the lemma, less often reaching 2/3 the length of the lemma; **rachilla** usually prolonged from the base of the floret (sometimes lacking in a small number of spikelets within the inflorescence), 0.2–0.5 mm long, glabrous, smooth to scabrous; **anthers** 0.4–1 mm long.

**Figure 11.**
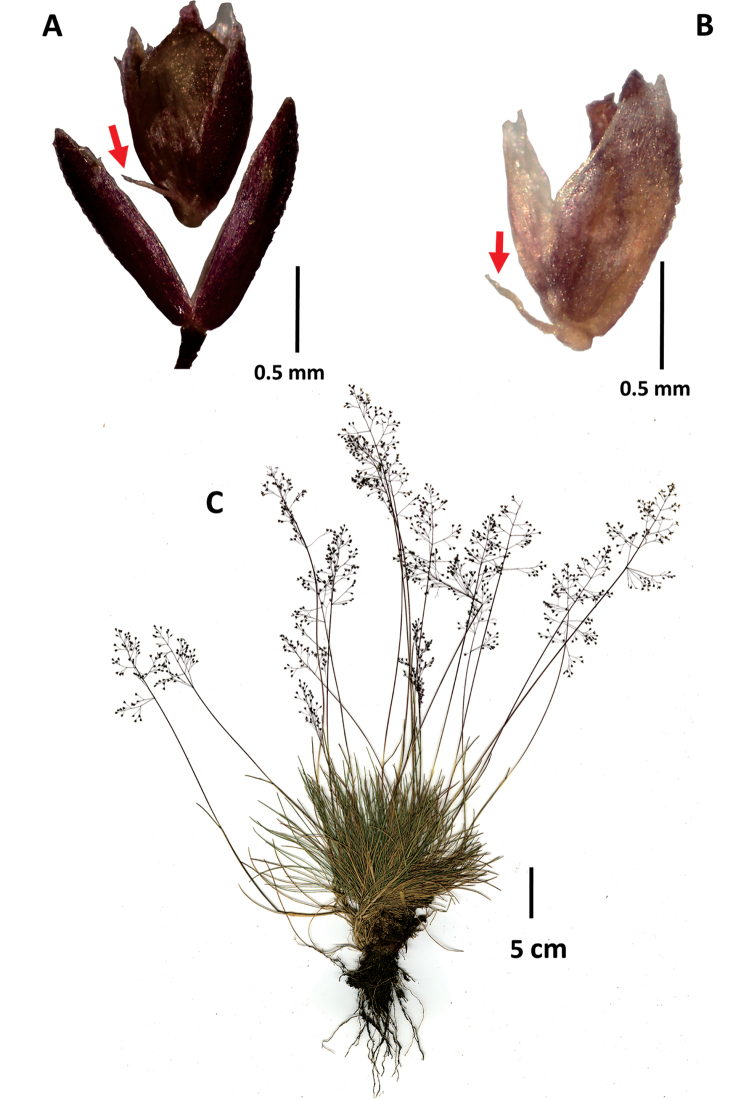
*Podagrostistrichodes***A** spikelet, lateral view with floret detached and raised above the glumes, rachilla prolongation indicated by an arrow **B** floret, lateral view, rachilla prolongation indicated by an arrow **C** whole plant. Images: **A**, **B** Rodríguez (UPTC 22204), **C** Cuta-Alarcon 362 (FMB).

##### Distribution and ecology.

Colombia, Ecuador?, Peru, Venezuela, 2800–4500 m alt. Relatively humid high-Andean puna grasslands of southern and central Peru and páramo grasslands of Ecuador, Colombia and Venezuela. May also occur in Bolivia according to Tovar (1993), although no specimens have been verified. No specimens have been verified from Ecuador, although it is mentioned to occur there (Hitchcock 1927; Tovar 1993; Jørgensen and Ulloa-Ulloa 1994; Jørgensen and León-Yánez 1999; Luteyn 1999). A common element in moderately grazed areas and path-sides of Boyacán páramo.

##### Other specimens examined.

See Sylvester et al. (2020).

##### Notes.

The combination of open panicle, spikelets < 1.5 mm long, florets which subequal the apex of the glumes, a palea reaching from (2/3) ¾ to subequaling the lemma, lemmas awnless or with a short (< 0.5 mm long) straight awn inserted in the upper ½ of the lemma, and a short glabrous rachilla prolongation emerging from under the palea are diagnostic for this species. The rachilla prolongation (Fig. 11, indicated by arrows) is sometimes difficult to see if it is tucked between the flanges of the palea, and so spikelet dissection is necessary. All species of *Podagrostis* from Colombia have involute or convolute leaf blades that can easily separate them from species of *Agrostis* with well-developed paleas.

##### Similar species.

Aside from *Podagrostisbacillata* (Hack.) Sylvester & Soreng and *P.exserta* (Swallen) Sylvester & Soreng that are found in Central America (see Sylvester et al. 2020), *P.trichodes* could possibly be mistaken for shorter plants of *A.perennans* which can have short spikelets as small as 1.8 mm long. *Agrostisperennans* s.l. has florets which usually do not reach past ¾ the length of the glumes, a minute palea less than ¼ the length of the lemma, and lacks a rachilla prolongation.

### Excluded species (from Boyacá, Colombia)

#### Agrostis gigantea

Taxon classificationPlantaePoalesPoaceae

Roth, Tent. Fl. Germ. 1: 31. 1788

6FD7B83B-1544-55AD-9CB0-1EC1A5B9A734

Agrostis vinealis var. gigantea (Roth) Willd., Sp. Pl., ed. 4, 1: 369. 1797.
Triticum giganteum (Roth) Roth, Catal. Bot. fasc. iii. 22. 1806.
Vilfa gigantea (Roth) P.Beauv., Ess. Agrostogr. 16. 1812.
Agrostis alba var. gigantea (Roth) Lej., Rev. Fl. Spa: 15. 1825.
Agrostis alba var. patula Klett & Richt., Fl. Leipzig: 71. 1830, nom. illeg.
Agrostis stolonifera var. gigantea (Roth) Rchb., Fl. Germ. Excurs.: 26. 1830.
Agrostis stolonifera subsp. gigantea (Roth) Schübl. & G. Martens, Fl. Würtemberg Ed. 1: 64. 1834.
Agrostis stolonifera var. gigantea (Roth) Bréb., Fl. Normandie 390. 1835.
Agrostis stolonifera var. gigantea (Roth) Klett & H. Richt. ex Peterm., Fl. Lips. Excurs. 83. 1838.
Agrostis stolonifera var. rothii Heuff., Enum. Pl. Banat.: 226. 1856, nom. superfl.
Agrostis signata var. gigantea (Roth) Schur, Oesterr. Bot. Z. 9: 48. 1859.
Agrostis alba subsp. gigantea (Roth) Arcang., Comp. Fl. Ital.: 768. 1882.
Agrostis stolonifera subsp. gigantea (Roth) Maire & Weiller, Fl. Afrique N.: 2(XLV): 120. 1953.
Agrostis stolonifera subsp. gigantea (Roth) Beldie, Fl. Republ. Socialist. Romania 12: 152. 1972.

##### Type.

Germany. [Inter arundinum et salices ad ripas Visurgis Ducatus Bremensis], A.W. Roth s.n. (lectotype, designated by Widén 1971: 97: G (G00195254 [image!]); isolectotypes: BM (BM001134107 [image!]), L).

Many heterotypic synonyms.

##### Notes.

Giraldo-Cañas et al. (2016) cite this species based on the species *Agrostistenuis* L. being recorded for Colombia by Hafliger and Scholz (1981). However, *A.tenuis* is a synonym of *A.capillaris*, and no specimens have been verified by us from either Departamento Boyacá or Colombia in general.

#### Agrostis lehmannii

Taxon classificationPlantaePoalesPoaceae

Swallen, Contr. U.S. Natl. Herb. 29 (6): 263. 1949

F316DE4C-BAEF-53AA-88EE-93500633110F

##### Type.

Colombia. Cauca: collected on páramo de Purace, Central Cordillera, 3500 m alt., 26 May 1944, E.P. Kilip & F.C. Lehmann 38598 (holotype: US (US1856227)).

##### Notes.

Only known from the type that was collected in the Cordillera Central of Colombia, but which was out on loan at the time of writing this publication. The protologue states the species to have a dense but rather lax inflorescence to 3 cm wide, florets lacking any notable palea, and a very long (c. 6 mm) awn inserted medially on the lemma dorsal surface. The length of the awn and ligule seems to differentiate this from *A.foliata* and *A.tolucensis*. This may be an odd *A.mertensii*, which is known to have panicle branches that can be appressed and congested when young while open when mature, although the ligule and awn length are all slightly past the limit for this species.

#### Agrostis scabrifolia

Taxon classificationPlantaePoalesPoaceae

Swallen, Contr. U.S. Natl. Herb. 29 (6): 264. 1949

C7F8480B-2256-5A52-A016-705333DB395B

##### Type.

Colombia. Norte de Santander: Collected on páramo de Tama, above Cueva, 3100–3200 m alt., 27 Oct. 1941, J. Cuatrecasas, R.E. Schultes, & E. Smith 12608 (holotype: US (US1850358)).

##### Notes.

Only known from the type, which was collected in the Cordillera Oriental, in Departamento Norte de Santander that is not too far from Departamento Boyacá and so may possibly occur there. The holotype was out on loan at the time of writing this publication to help clarify its identity. The open panicle and spikelets with palea minute or absent and lemmas with a well-developed dorsally inserted awn are very distinct characteristics which would place the species close to *A.mertensii*. The species is mentioned to have scabrous culms, at least below the nodes, scaberulous sheaths and scabrous leaf blades while *A.mertensii* is generally smooth or only scaberulous in the leaf blade adaxial surface and margin. The leaf blades are also mentioned to be stiff and erect while those of *A.mertensii* are generally lax and soft.

#### Agrostis subrepens

Taxon classificationPlantaePoalesPoaceae

(Hitchc.) Hitchc., N. Amer. Fl. 17 (7): 525. 1937

3AE720BF-0D3C-5BE6-94C0-B31295CB6E1C

Agrostis hiemalis var. subrepens Hitchc., U.S.D.A. Bur. Pl. Industr. Bull. 68: 44. 1905.
Agrostis hyemalis var. subrepens Hitchc., U.S.D.A. Bur. Pl. Industr. Bull. 68: 44. 1905.

##### Type.

Mexico. Chihuahua: in wet places, pine plains, base of Sierra Madre Mountains, 28 Sep. 1887, C.G. Pringle 1420 (holotype: US (US00131756 [image!]); isotypes: BAB (BAB00000208 [image!] fragm. ex US), E (E00381793 [image!]), F (F0046565F [image!]), GH (GH00221377 [image!]), K (K000308371 [image!]), MSC (MSC0129855 [image!]), NY (NY00327645 [image!], NY00327646 [image!]), US (US00131757 [image!])).

##### Other specimens examined.

**Mexico**. **Chihuahua**: [Munic. Casas Grandes], near Colonia García in the Sierra Madre, 1 Aug. 1899, E.W. Nelson 6195(US359911); Chichupa, 23 Aug. 1937, Harde LeSueur 198 (US1721671).

**Venezuela**. Sin loc., no date, Fendler 2541 (US843224).

##### Notes.

Ambiguity surrounds the identity and placement of *A.subrepens*. Hitchcock (1905) initially described this as a variety of *A.hyemalis* (Walter) Britton, Sterns & Poggenb., but then raised it to the level of species 32 years later. Originally stated to occur in northern Mexico, and the states New Mexico, Nevada, Arizona of the USA, and Venezuela (Hitchcock 1905), in his later account, Hitchcock (1937) then restricts the distribution to wet places of the Sierra Madre, Chihuahua, Mexico, and also Venezuela based on the specimen Fendler 2541 (US843224). This Venezuelan paratype was seen by us at US but only comprised two fragments of an inflorescence and no leaves. As both *A.perennans* and *A.subrepens* have strikingly similar spikelet morphology, there was no way to be confident on its placement, raising ambiguity over whether *A.subrepens* occurs in South America. The species has been included in checklists or taxonomic treatments for Bolivia (Jørgensen et al. 2014), Colombia (Giraldo-Cañas et al. 2016), Peru (Tovar 1993), and Venezuela (Hokche et al. 2008). The identification key and description of *A.subrepens* in the treatment for Peru (Tovar 1993) do not match the type specimens and it may be that Tovar (1993) had not seen types as he distinguishes this from *A.perennans* (sub *A.humboldtiana*) and *A.imberbis* (sub *A.stenophylla*) in part by the leaf blades being flat and flaccid. Although Giraldo-Cañas et al. (2016) mention this species to occur in Departamentos Boyacá, Cundinamarca, and Santander of the Cordillera Oriental, and Antioquia, Huila, and Quindio of the Cordillera Central, no specimens have been verified by us, although we did not revise specimens outside of Boyacá while at COL and none were verified from these provinces while at US. While we have found certain characteristics to differentiate this from *A.imberbis*, such as culm and panicle size, and ligule form, it may be that these are the same and that *A.imberbis* is a species more amply distributed. See comments under Agrostiscf.imberbis and *A.perennans* s.l. for how to differentiate these from *A.subrepens*.

#### Agrostis turrialbae

Taxon classificationPlantaePoalesPoaceae

Mez, Repert. Spec. Nov. Regni Veg. 18 (1–3): 4. 1922

4316A902-9615-59B5-BC7A-CB2D8730E969

= Agrostisarcta Swallen, Contr. U.S. Natl. Herb. 29 (9): 405. 1950. Type: Guatemala. Chimaltenango: moist roadside at Santa Elena, 2400−2700 m alt., 17 July 1933, A.F. Skutch 422 (holotype: US (US00131720)).
= Agrostisvesca Swallen, Contr. U.S. Natl. Herb. 29 (9): 405. 1950. Type: Guatemala. Chimaltenango: collected on open, moist roadside at Santa Elena, 2400−2700 m alt., 17 July 1933, A.F. Skutch 420 (holotype: US (US00131129)).

##### Type.

Costa Rica. Cartago: Volcán Turrialba, 27 May 1884, H. Pittier 855 (holotype: B; isotype: US (US00131127)).

##### Specimen examined.

**Costa Rica**. San Jose: Along Interamerican Highway ca 8.5 km E of road to La Cima, approximately 9°40'N, 83°51'W, 2600−2650 m alt., roadside and below highway, remnant evergreen forest, 30 July 1979, W.D. Stevens 13370 (MO2820979 [image!]).

##### Notes.

Described from páramos of Costa Rica, and mentioned by Pohl and Davidse (1994) to also occur in alpine pastures of Guatemala and Mexico, *A.turrialbae* has also been included in checklists for Colombia (Giraldo-Cañas et al. 2016) and Venezuela (Hokche et al. 2008). However, there is a lack of consensus on the identity of *A.turrialbae* and how to differentiate it from *A.perennans* s.l., with all type material of *A.turrialbae* and its synonyms out on loan at the time of writing this publication to help clarify this. The principal discrepancy refers to the form of the leaf blades, with the protologue and Pohl (1980) mentioning filiform flat or folded leaf blades, while Pohl and Davidse (1994) and Morales-Quirós (2003) state the blades to be involute and narrow, 0.2−1 mm in diameter as rolled, and rarely the cauline leaf blades being flat, and use this character to differentiate it from the usually flat bladed *A.perennans*. This may relate to the species *A.arcta* and *A.vesca*, described from Guatemala and considered synonyms of *A.turrialbae* by Pohl and Davidse (1994), with the protologues mentioning leaf blades being firm, folded and involute, curved, in *A.arcta*, or flat, filiform, or involute and straight in *A.vesca. Agrostisperennans*, in its broad sense, can also have involute or filiform leaf blades based on species currently considered synonyms of *A.perennans*, e.g. *A.aberrans* Steud., *A.kufium* Speg., A.tenuifoliaM. Bieb.var.fretensis Hook. f., *Vilfaelegans*.

While all literature (Pohl 1980; Pohl and Davidse 1994; Morales-Quirós 2003) also differentiates *A.perennans* from *A.turrialbae* by stating there is a lack of conspicuous basal foliage in the former, this may not be a good differentiating character as *A.perennans* usually has blades concentrated at the base of the plant when it is young, and only in subsequent flowering seasons it begins to elongate and lose its basal foliage. Pohl (1980) also mentions that paleas are minute in *A.perennans*, while in *A.turrialbae* paleas are absent, but this character appears to be too variable to be useful. Discrepancy in spikelet size is also apparent, with the *A.turrialbae* protologue stating 1.75 mm long and the *A.vesca* protologue stating 1.6−1.8 mm long, while all other publications describing the species (Pohl 1980; Pohl and Davidse 1994; Morales-Quirós 2003) state 2−2.1 mm or 2−2.8 mm long.

We here tentatively differentiate the two species by *A.turrialbae* having leaf blades filiform, 0.2−1 mm wide when opened out, thin and flaccid, leaves mainly basal at maturity, and plants generally smaller with culms to 40 cm tall and panicles to 12 cm long and 6 cm wide (vs. leaf blades flat or conduplicate, (1–)1.5–6 mm wide when opened out, rarely involute in the basal leaves, usually thickened at the margins and keel, firm, leaves mainly basal early in the flowering season but tending to become mostly cauline with maturity, and plants generally larger, with culms to 100 cm tall and panicles often larger, to 22 cm long and 11 cm wide in *A.perennans* s.l.). No specimens of *A.turrialbae* that fit this delineation have been found at US from either Colombia or Venezuela. Furthermore, *A.turrialbae* may be better included among the broad *A.perennans* complex until a satisfactory revision can be done of this species complex, which has been found to comprise evolutionarily distinct lineages in unpublished molecular phylogenies (Konstantin Romaschenko, pers. communication).

## Supplementary Material

XML Treatment for Agrostis

XML Treatment for Agrostis boyacensis

XML Treatment for Agrostis breviculmis

XML Treatment for Agrostis capillaris

XML Treatment for Agrostis foliata

XML Treatment for Agrostis cf. imberbis

XML Treatment for Agrostis mertensii

XML Treatment for Agrostis perennans

XML Treatment for Agrostis stolonifera

XML Treatment for Agrostis tolucensis

XML Treatment for Podagrostis

XML Treatment for Podagrostis trichodes

XML Treatment for Agrostis gigantea

XML Treatment for Agrostis lehmannii

XML Treatment for Agrostis scabrifolia

XML Treatment for Agrostis subrepens

XML Treatment for Agrostis turrialbae
